# DGF-YOLO: A Degradation-Guided Feature Enhancement Method for Small-Scale Pedestrian Detection in UAV Images

**DOI:** 10.3390/jimaging12090413

**Published:** 2026-09-02

**Authors:** Boyu Wang, Jingguo Lv, Shuwei Huang, Yingqi Bai

**Affiliations:** 1 School of Geomatics and Urban Spatial Information, Beijing University of Civil Engineering and Architecture, Beijing 100044, China; 2108160224009@stu.bucea.edu.cn (B.W.); 2108160224008@stu.bucea.edu.cn (S.H.); 2 SpaceWill Info. Co., Ltd., Beijing 100089, China; yingqi.bai@spacewillinfo.com

**Keywords:** UAV imagery, small-object detection, pedestrian detection, structural degradation

## Abstract

Small-scale pedestrians in UAV imagery often exhibit limited pixel coverage, weak texture, and severe background interference, while progressive network downsampling can further degrade their short-side structures and increase missed detections. To address this problem, we propose DGF-YOLO, a degradation-guided feature enhancement method built on YOLOv12n. The method introduces a degradation-level criterion to identify the feature stage at which a pedestrian first undergoes significant structural degradation and, based on the resulting statistics, incorporates a high-resolution P2 detection head. It further employs a Directional Structure-Aware module to enhance local, horizontal, and vertical structural cues through adaptive multi-branch fusion, a Degradation-Guided Attention module to learn a degradation guidance map under explicit supervision and reweight degradation-sensitive regions, and a Fine-Grained Structure Preservation module to retain local contours and contextual details using depthwise and dilated convolutions. On the single-class pedestrian detection task constructed from VisDrone2019-DET, DGF-YOLO achieves 70.4% precision, 51.8% recall, 59.7% mAP50, and 26.9% mAP50-95, improving the YOLOv12n baseline by 6.6, 7.4, 10.3, and 6.5 percentage points, respectively. The results suggest that the proposed feature enhancement strategy helps reduce missed detections associated with structural degradation in small-scale pedestrians.

## 1. Introduction

With the rapid development of the low-altitude economy, unmanned aerial vehicles (UAVs) have become important aerial platforms for smart city management, public-safety inspection, and emergency situational awareness because of their high mobility, wide coverage, low deployment cost, and flexible viewpoints. Pedestrian detection in UAV-captured aerial images is a fundamental component of applications such as public-security surveillance, large-event security, disaster rescue, and crowd counting, and therefore has considerable practical and research significance. However, compared with images acquired by fixed surveillance cameras, UAV imagery is typically captured from higher viewpoints, covers larger areas, and contains more complex nadir or oblique perspectives. Consequently, pedestrians often exhibit limited pixel coverage, weak texture, substantial scale variation, and severe background interference. In medium- and low-altitude inspection and large-area monitoring scenarios, the effective pixel size of a pedestrian is jointly determined by flight altitude, camera focal length, sensor size, and ground sampling distance (GSD), making pedestrians prone to appearing as small-scale targets with weak structural cues.

Taking the DJI Mavic 3E, a representative UAV for low-altitude inspection, as an example, its wide-angle camera is equipped with a 4/3-inch CMOS sensor, provides 20 MP effective pixels, and supports a maximum image resolution of 5280 × 3956 pixels [1]. According to the physical imaging parameters of the camera and the GSD calculation model, the corresponding GSD is approximately 1.75–3.50 cm/pixel at flight altitudes of 64–128 m [2,3]. Referring to the lower-percentile shoulder-breadth statistics reported in GB/T 10000—2023, Human Dimensions of Chinese Adults, the pedestrian shoulder width is approximated as 0.35 m in this study [4]. Under these imaging conditions, the short side corresponding to the shoulder width generally occupies only approximately 10–20 pixels in the input image. After progressive downsampling at the P3 and P4 levels of YOLO-based detection networks, this short-side structure is compressed to approximately 1–3 feature cells, or even fewer than one cell. In such cases, the head–shoulder contour, torso boundary, and local directional structures cannot be adequately represented in the feature maps. Low-resolution pedestrians may therefore be confused with background patterns such as road textures, shadows, and pole-like objects, leading to a large number of missed detections. The above GSD analysis is intended to explain the physical origin of short-side degradation in small-scale pedestrians under typical low-altitude UAV imaging conditions. Under oblique viewing angles, perspective projection and foreshortening may alter the apparent width and height of pedestrians; therefore, the shoulder-width approximation is used only to provide a physical interpretation of short-side degradation rather than to determine the degradation state of individual targets. VisDrone2019-DET is a challenging UAV-based object detection benchmark containing ten object categories captured under diverse urban scenes, viewing conditions, and imaging environments. The dataset contains numerous small-scale and densely distributed objects with substantial scale variation, occlusion, and complex background interference, making it well suited for evaluating object detection methods in UAV imagery [5]. Because VisDrone2019 does not provide unified camera parameters or flight-altitude information, the degradation states of pedestrian targets are subsequently analyzed using the short-side dimensions of annotated bounding boxes and the downsampling strides of the network.

Deep learning-based object detection has developed along two-stage and one-stage paradigms. The R-CNN [6] series progressively integrated CNN feature learning, region classification, and proposal generation, with Faster R-CNN [7] introducing the Region Proposal Network (RPN) to establish a representative two-stage detection framework. In contrast, YOLO [8] formulated object detection as a unified end-to-end regression problem and laid the foundation for efficient one-stage detectors. Building on these fundamental frameworks, recent small-object detection studies have mainly focused on input-scale enhancement, high-resolution feature representation, target feature enhancement, and training-supervision optimization. Akyon et al. proposed SAHI, which increases the relative scale of small objects at the network input through overlapping image slicing and improves small-object detection performance on aerial datasets such as VisDrone and xView [9]. Yang et al. introduced QueryDet, which employs a sparse-query mechanism to locate potential small-object regions on low-resolution feature maps and then performs fine-grained local detection on high-resolution features [10]. Liu et al. proposed ESOD, which combines feature-level object search with local slicing to allocate computational resources to regions more likely to contain small objects, thereby balancing detection accuracy and computational efficiency [11]. For high-resolution feature representation, Hu et al. introduced shallow high-resolution feature maps together with detail-guidance and feature-refinement modules to strengthen edge, texture, and local structural representations of small objects [12]. Chen et al. proposed HR-FPN, which reduces the loss of small-object details during downsampling through high-resolution feature alignment and cross-level fusion, thereby improving feature representation quality [13]. In addition, SR-TOD [14], pixel-level information-guided methods [15], and the spectral enhancement framework SET [16] improve the separability between tiny objects and complex backgrounds from the perspectives of spatial discrepancy, pixel information, and frequency-domain enhancement, respectively. RFLA [17], DCFL [18], and SimD [19] instead optimize label assignment and training supervision to increase the probability that tiny objects receive effective supervisory signals.

Most existing methods improve object representation through input rescaling, feature fusion, attention enhancement, or label assignment, whereas the structural degradation of targets during progressive network downsampling has received comparatively limited attention. Moreover, small objects are commonly treated as a general scale-related problem, while the directional degradation characteristics of UAV-captured pedestrians, jointly caused by limited imaging resolution, elongated morphology, and weak structural appearance, are often overlooked. When short-side structures such as shoulder width occupy only 10–20 pixels in the input image, they are rapidly compressed to only a few feature cells after downsampling at detection levels such as P3 and P4. As a result, head–shoulder contours, body boundaries, and local directional structures are difficult to represent effectively. Therefore, the key to detecting small-scale pedestrians in UAV imagery lies not only in improving their overall scale representation, but also in identifying the feature level at which structural degradation occurs and enhancing directional structures and local details either before severe degradation or within degradation-sensitive regions.

To address these issues, this study proposes DGF-YOLO, a degradation-guided feature enhancement method for small-scale pedestrian detection in UAV imagery. First, the concept of a degradation level is introduced based on the relationship between the short-side pixel dimension of a target and the downsampling stride of the network. This concept identifies the feature level at which a pedestrian first undergoes significant structural degradation and provides a more explicit criterion for analyzing the difficulty of small-scale pedestrian detection. Second, according to the statistical distribution of degradation levels, a high-resolution P2 detection head is incorporated into YOLOv12. This design enables small-scale pedestrians to retain richer spatial representations before severe short-side degradation occurs and alleviates the insufficient local structural information available at the conventional P3 detection level. Furthermore, considering the elongated morphology, short-side vulnerability, and weak local details of UAV-captured pedestrians, a degradation-aware feature enhancement mechanism is developed. It comprises a Directional Structure-Aware module, a Degradation-Guided Attention module, and a Fine-Grained Structure Preservation module, which enhance low-resolution pedestrian features through directional structure modeling, degradation-region enhancement, and local-detail preservation, respectively. The main contributions of this study are summarized as follows:

1. This study introduces the concept of degradation level for UAV-based pedestrian detection. By examining the relationships among GSD, target short-side pixel dimensions, and network downsampling strides, the proposed criterion identifies the feature level at which a pedestrian first undergoes significant structural degradation. This establishes a link between the input imaging scale and the internal representation state of the network, providing a new perspective for analyzing missed detections of small-scale pedestrians.
2. Based on the statistical distribution of degradation levels, a high-resolution P2 detection head is incorporated into YOLOv12n, providing a task-specific basis for selecting the shallow detection scale. This design enables more small-scale pedestrians to retain richer spatial and structural representations before severe short-side degradation occurs, thereby alleviating missed detections caused by insufficient feature representation at the conventional P3 level.
3. A degradation-aware feature enhancement mechanism comprising the Directional Structure-Aware, Degradation-Guided Attention, and Fine-Grained Structure Preservation modules is developed to model directional structures, degradation-sensitive regions, and local details, respectively.

The method is evaluated through ablation studies, degradation-level-specific recall analysis, comparisons with existing detectors, and qualitative visualization on the VisDrone2019 pedestrian detection task.

## 2. Materials and Methods

**Figure 1:**
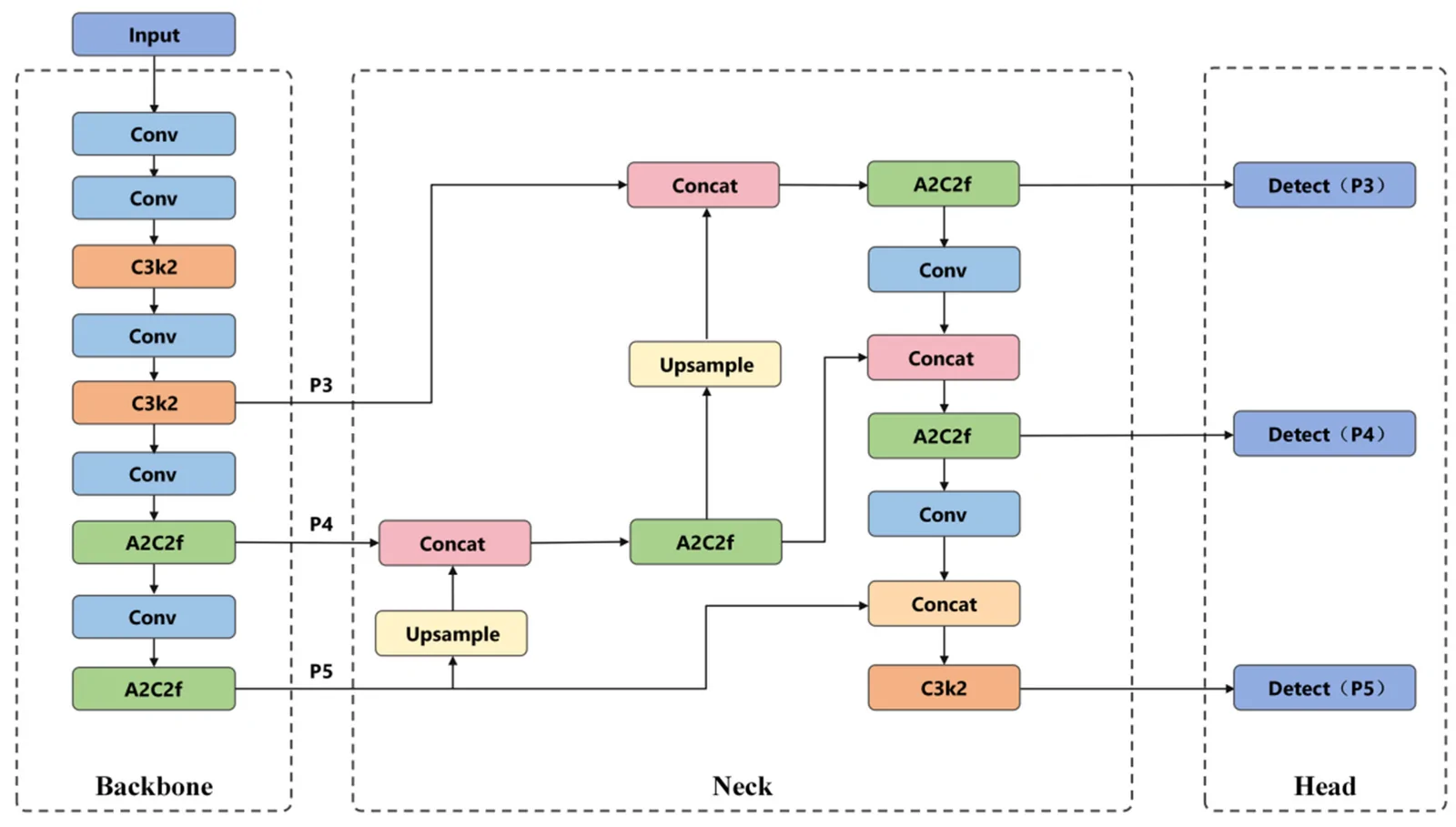
Network architecture of YOLOv12.

This study adopts YOLOv12 [20] as the baseline detector and introduces targeted modifications to its feature extraction and multi-scale detection architecture, considering that pedestrian targets in UAV aerial imagery are prone to structural degradation during successive network downsampling. The overall architecture of YOLOv12 is illustrated in Figure 1. The model mainly consists of three components: a backbone, a neck, and a detection head. The input image is first resized to a predefined resolution and then fed into the backbone for hierarchical feature extraction. While retaining the efficient one-stage detection framework of the YOLO family, YOLOv12 introduces the Area Attention mechanism (A2). This mechanism partitions the feature map into multiple local regions and performs attention computation within each region, thereby effectively reducing the computational overhead associated with conventional self-attention while preserving a large receptive field and the capability to model global contextual information. In addition, YOLOv12 employs Residual Efficient Layer Aggregation Networks (R-ELAN), which enhance information propagation through residual connections and inter-layer feature aggregation. This design facilitates gradient flow during deep-network training and improves the representational quality of multi-scale features. During the feature fusion stage, the neck performs both top-down and bottom-up information interaction among feature maps at different scales generated by the backbone. In this manner, the strong semantic information contained in deep features is integrated with the rich spatial details preserved in shallow features, producing hierarchical feature representations suitable for detecting objects at different scales. Finally, the detection head performs bounding-box regression and category prediction based on the fused multi-scale feature maps, enabling unified detection of large-, medium-, and small-scale objects. YOLOv12 achieves a favorable balance between real-time detection efficiency and feature modeling capability. Its area attention mechanism and layer aggregation architecture provide a strong representational foundation for object recognition in complex scenes. Therefore, YOLOv12 is selected as the baseline model for the UAV-based pedestrian detection task in this study.

However, the original YOLOv12 architecture primarily follows a conventional multi-scale detection paradigm, in which the default detection layers are designed to balance computational efficiency and scale adaptability. For small-scale pedestrians in UAV aerial imagery, their low pixel occupancy and limited short-side dimensions make their contours, boundaries, and local directional structures susceptible to progressive compression or even structural degradation during successive network downsampling. Consequently, the conventional P3 detection layer may fail to preserve sufficient spatial structural information, thereby exacerbating missed detections during inference.

**Figure 2:**
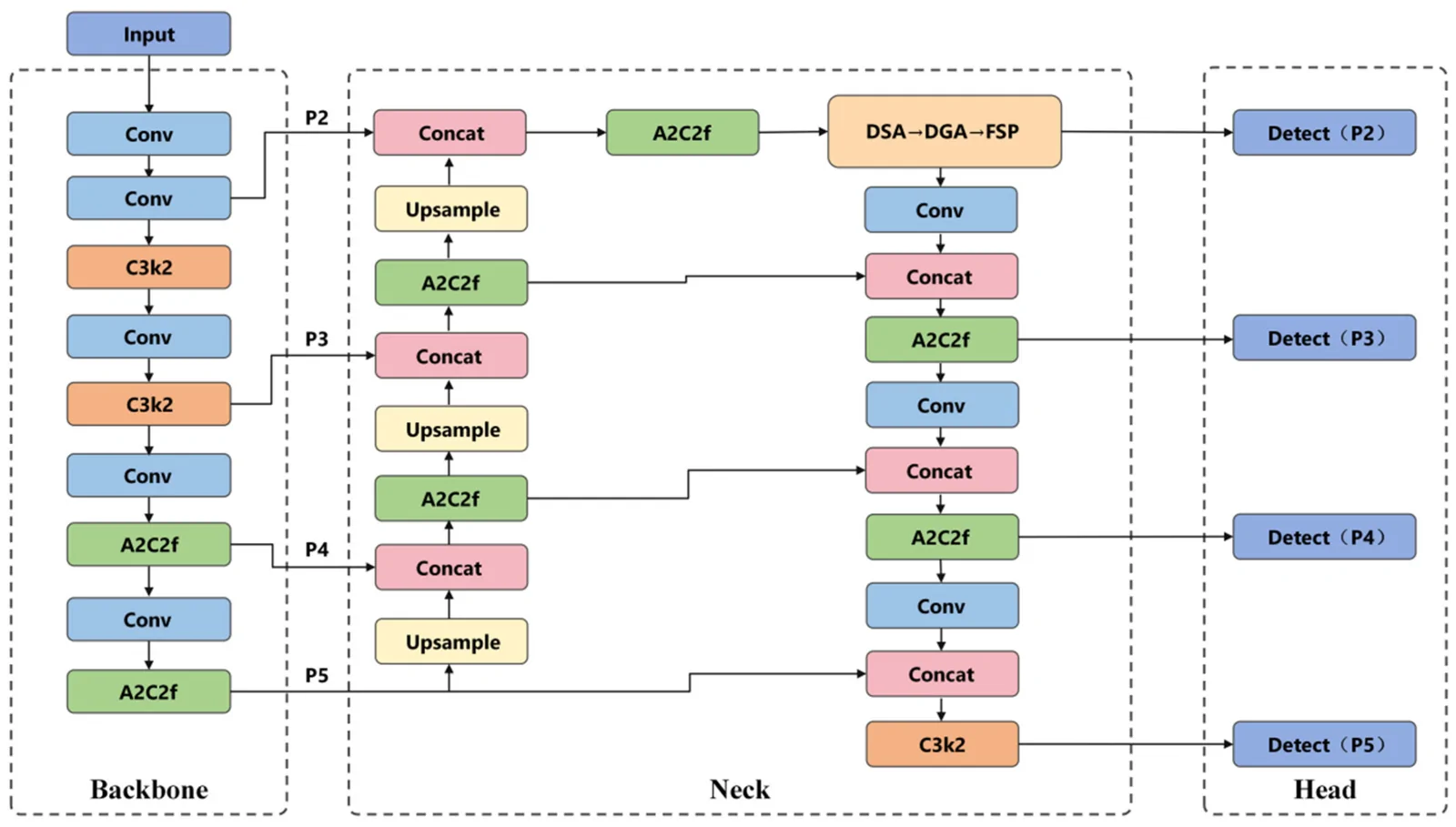
Overall architecture of DGF-YOLO.

This study proposes a degradation-guided feature enhancement method for small-scale pedestrian detection, termed DGF-YOLO (Degradation-Guided Feature Enhancement YOLO). Motivated by the structural degradation of small-scale pedestrians during successive downsampling in UAV aerial imagery, the proposed method performs targeted modeling of spatial details, directional structures, and degradation-sensitive regions. First, a high-resolution P2 detection layer is incorporated into the original multi-scale detection architecture of YOLOv12, extending small-scale pedestrian detection to a higher-resolution feature level. This design preserves object contours, boundaries, and local details at an earlier stage, thereby alleviating the structural degradation caused by network downsampling. On this basis, a degradation-aware feature enhancement mechanism is constructed, consisting of a Directional Structure-Aware Module (DSA), a Degradation-Guided Attention Module (DGA), and a Fine-Grained Structure Preservation Module (FSP). Specifically, DSA addresses the imbalanced aspect ratios and short-side degradation of pedestrian targets by employing parallel local, horizontal, and vertical branches to enhance directional structure representation. DGA strengthens the features of degradation-sensitive target regions through degradation-guided attention, thereby improving the discriminability between small-scale pedestrians and complex backgrounds. FSP further preserves local contours, boundaries, and contextual information, enhancing the fine-grained structural representation of degraded small-scale pedestrians. Through these designs, DGF-YOLO combines high-resolution P2 detection with directional modeling, degradation-guided spatial reweighting, and local-detail preservation.The overall architecture of DGF-YOLO is illustrated in Figure 2.

### 2.1. Degradation Level

Existing definitions of small objects are generally based on their pixel dimensions in the input image [21,22] or the ratio of the object area to the total image area [23,24]. Although these definitions characterize object size at the image scale, they cannot adequately reflect changes in structural representation during successive downsampling within a detection network. For pedestrian targets in UAV aerial imagery, the imaging scale is determined not only by their physical dimensions but also by factors such as flight altitude, camera focal length, sensor size, and ground sampling distance (GSD). As the GSD increases, the effective pixel dimensions of pedestrians in the input image decrease substantially. In particular, short-side structures, such as the transverse shoulder width, are more likely to be represented as weak structures with limited pixel support.

Let $W_{real}$ denote the physical short-side width of a pedestrian target, GSD denote the ground sampling distance of the aerial image, and $W_{pix}$ denote the short-side dimension of the target in pixels in the input image. Their relationship can be expressed as

(1) $$W_{pix}=\frac{W_{real}}{GSD},$$

$W_{real}$ can be approximated by the biacromial breadth defined in anthropometry. As indicated by Equation (1), when the physical short-side width of a pedestrian remains relatively constant, a larger GSD results in a smaller short-side dimension in pixels in the input image, thereby increasing the risk of structural degradation in subsequent feature maps.

Let w and h denote the width and height of the target bounding box in the network input image, respectively. Let $s_{l}$ denote the downsampling stride of the l-th feature level relative to the input image, and let $w_{l}$ and $h_{l}$ denote the width and height of the target after being mapped onto this feature level, respectively. Their relationship can be expressed as

(2) $$w_{l}=\frac{w}{s_{l}},h_{l}=\frac{h}{s_{l}}$$

$w_{l}$ and $h_{l}$ denote the mapped width and height of the target at the l-th feature level, respectively. As the network depth increases, the downsampling stride becomes progressively larger, continuously reducing the spatial extent of the target on the feature maps. For elongated objects such as pedestrians, the short-side dimension is typically compressed to only a few feature units earlier than the long-side dimension. Therefore, the mapped short-side dimension provides a more representative measure of the target’s structural preservation state.

The mapped short-side dimension of the target at the l-th feature level is denoted by $d_{l}$ and defined as

(3) $$d_{l}=min(w_{l},h_{l})=\frac{min(w,h)}{s_{l}}$$

**Figure 3:**
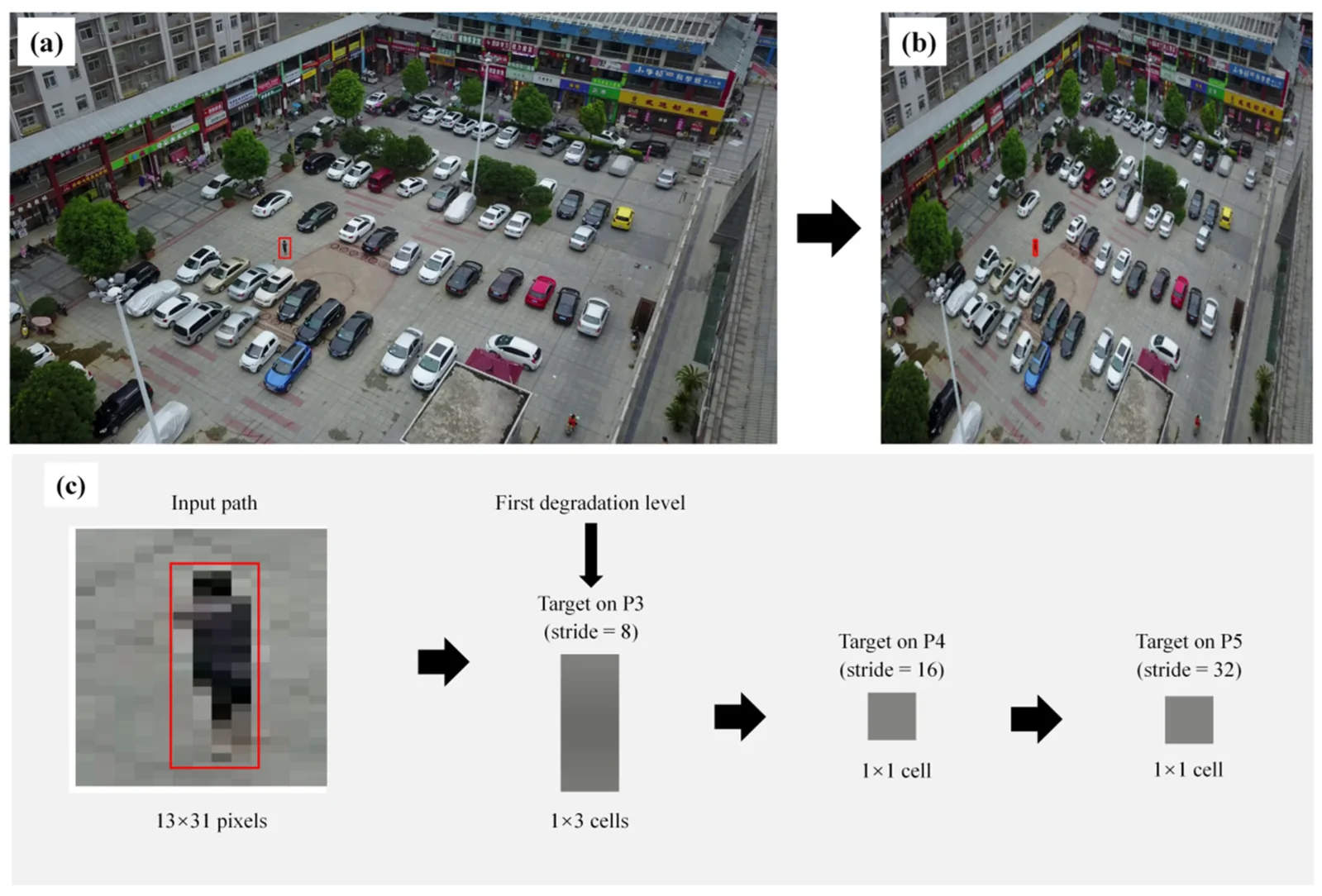
Schematic illustration of the structural degradation of small-scale pedestrians during successive downsampling. (**a**) Original UAV aerial image (1920 × 1080); (**b**) network input image (640 × 640); (**c**) target mappings at different feature levels. The red bounding boxes indicate the pedestrian targets used for subsequent structural degradation analysis.

Because $d_{l}$ is calculated from the observed bounding-box dimensions in the input image, variations in apparent target size caused by viewing angle, perspective projection, and pedestrian pose are implicitly reflected in this feature-space measure. Combining Equations (2) and (3), when the mapped short-side dimension satisfies $d_{l}\leq 1$, the target extent along the short-side direction has been reduced to no more than one feature unit after successive scale mappings. Under this condition, the head–shoulder contour, body boundaries, and local directional structures of a pedestrian can no longer be jointly represented by multiple adjacent feature units, indicating substantial compression of its spatial structural information. In this study, the feature level at which the mapped short-side dimension first satisfies $d_{l}\leq 1$ is defined as the degradation level of the target. To intuitively illustrate the structural degradation of small-scale pedestrians during successive network downsampling in UAV aerial imagery, a representative example is presented in Figure 3.

As shown in Figure 3, after the pedestrian target in the original aerial image is resized to the network input resolution, its pixel information is further compressed, resulting in a low-pixel, elongated structural representation. As the stride of the feature levels increases, the spatial extent of the target on the feature maps is progressively reduced, with the short-side structure typically degrading earlier than the long-side structure. In this example, the mapped short-side dimension of the target is reduced to one feature unit for the first time at the P3 level. Therefore, P3 is defined as its initial degradation level. At the deeper P4 and P5 levels, the target is further compressed into a single feature unit, retaining only limited target representation and losing additional local structural information.

According to the feature level at which structural degradation first occurs, the targets are categorized into four groups: P3-degraded, P4-degraded, P5-degraded, and non-degraded targets. Specifically, a P3-degraded target first satisfies the single-feature-unit degradation criterion along its short side at the P3 level. A P4-degraded target retains a certain degree of short-side structure at P3 but begins to undergo structural degradation at P4. A P5-degraded target exhibits evident structural degradation only at a deeper feature level. A non-degraded target does not satisfy the single-feature-unit degradation criterion at or before the P5 level. In general, an earlier degradation level indicates that the target loses its fine-grained spatial structure earlier during network downsampling, making it more difficult to maintain an effective representation during deep semantic modeling.

**Figure 4:**
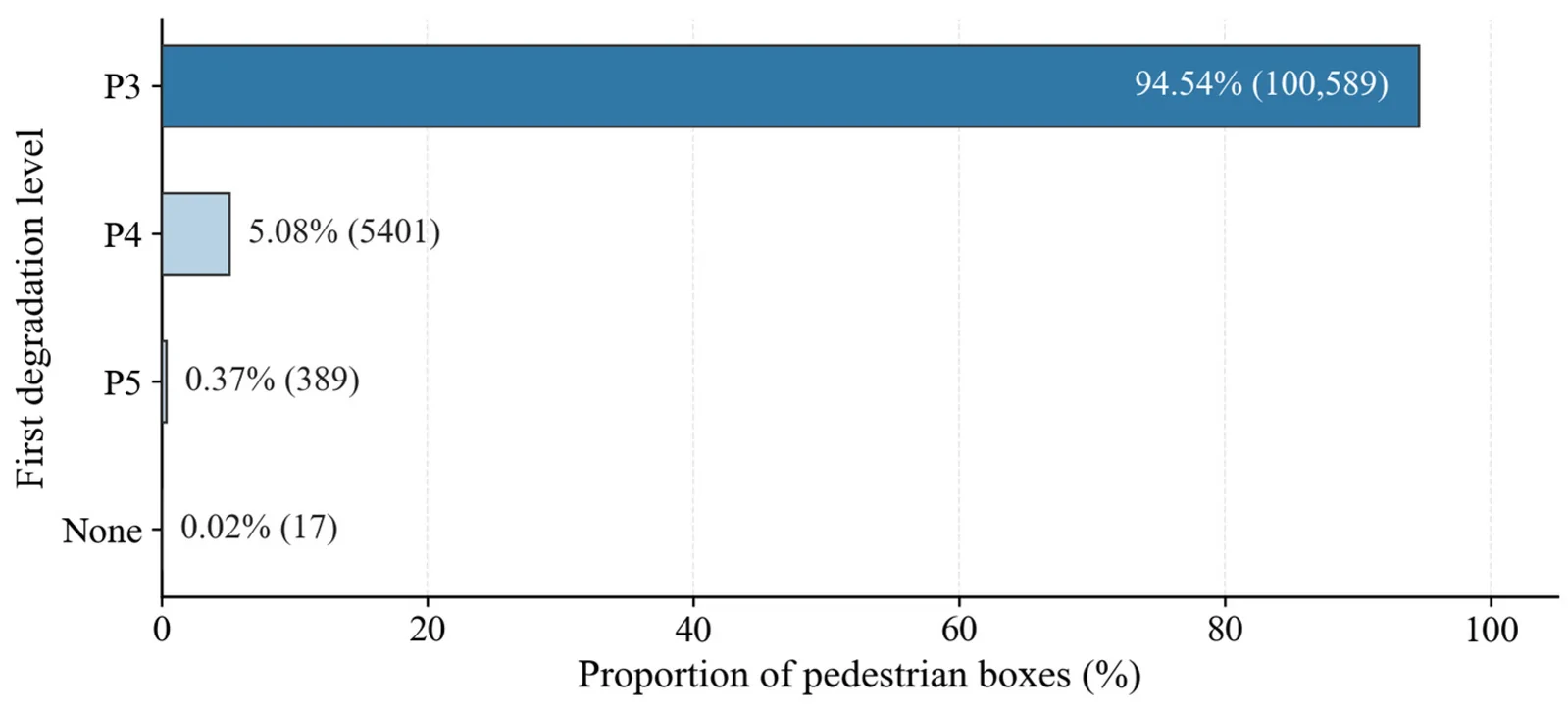
Distribution of the initial degradation levels of pedestrian targets in the VisDrone2019-DET Training Set.

Based on the above definition, the initial degradation levels of pedestrian targets in the VisDrone2019 training set were statistically analyzed. The analysis covered 6471 annotation files and 106,396 pedestrian bounding boxes, and the results are presented in Figure 4.

The results show that 100,589 targets are categorized as P3-degraded, accounting for 94.54% of all pedestrian instances. The numbers of P4-degraded, P5-degraded, and non-degraded targets are 5401, 389, and 17, corresponding to 5.08%, 0.37%, and 0.02%, respectively. According to the statistics of the mapped short-side dimensions, the conventional P3 feature level cannot provide a multi-feature-unit representation along the short-side direction for the majority of pedestrians in UAV aerial imagery. This limitation may constitute an important cause of missed detections of small-scale pedestrians.

To further investigate whether higher-resolution features can alleviate structural degradation, the P2 feature level was incorporated into the degradation-level analysis. The results show that 71.49% of the targets undergo short-side degradation at P2, representing a reduction of 23.05 percentage points compared with the 94.54% observed at P3. Although a substantial proportion of extremely small pedestrians still undergo short-side degradation at P2, the higher spatial resolution of P2 enables more targets to retain a certain degree of spatial structure than P3. Moreover, even for targets that satisfy the degradation criterion, P2 provides relatively richer representations of their contours, boundaries, and local details. Therefore, a high-resolution P2 detection layer is introduced into the original P3/P4/P5 multi-scale detection architecture of YOLOv12, extending small-scale pedestrian detection to a shallower feature level to alleviate structural degradation caused by network downsampling.

### 2.2. Degradation-Aware Feature Enhancement Mechanism

As indicated by the degradation-level analysis in Section 2.1, pedestrian targets in UAV aerial imagery generally exhibit short-side structural degradation at the conventional P3 feature level, whereas the high-resolution P2 feature level can preserve more complete spatial structural information to a certain extent. However, for small-scale pedestrians that have already undergone or are close to undergoing structural degradation, relying solely on a high-resolution detection layer remains insufficient to fully recover their directional structures, boundary contours, and local details. Therefore, the proposed method does not simply depend on high-resolution detection to improve small-object detection performance, but further performs targeted feature enhancement according to the characteristics of structural degradation.

To this end, a degradation-aware feature enhancement mechanism is developed on the basis of the degradation-level analysis, enabling the network to explicitly model the structural degradation characteristics of small-scale pedestrians. Specifically, to address short-side structural compression, insufficient discriminability of degradation-sensitive regions, and weakened fine-grained structural information during network downsampling, the proposed mechanism consists of a Directional Structure-Aware Module (DSA), a Degradation-Guided Attention Module (DGA), and a Fine-Grained Structure Preservation Module (FSP). DSA enhances the representation of horizontal and vertical structures of pedestrian targets, DGA strengthens the target features in degradation-sensitive regions, and FSP preserves local contours, boundaries, and contextual details. Through the coordinated operation of these modules, the network further improves the structural representation of degraded small-scale pedestrians based on the high-resolution P2 features.

#### 2.2.1. Directional Structure-Aware Module

**Figure 5:**
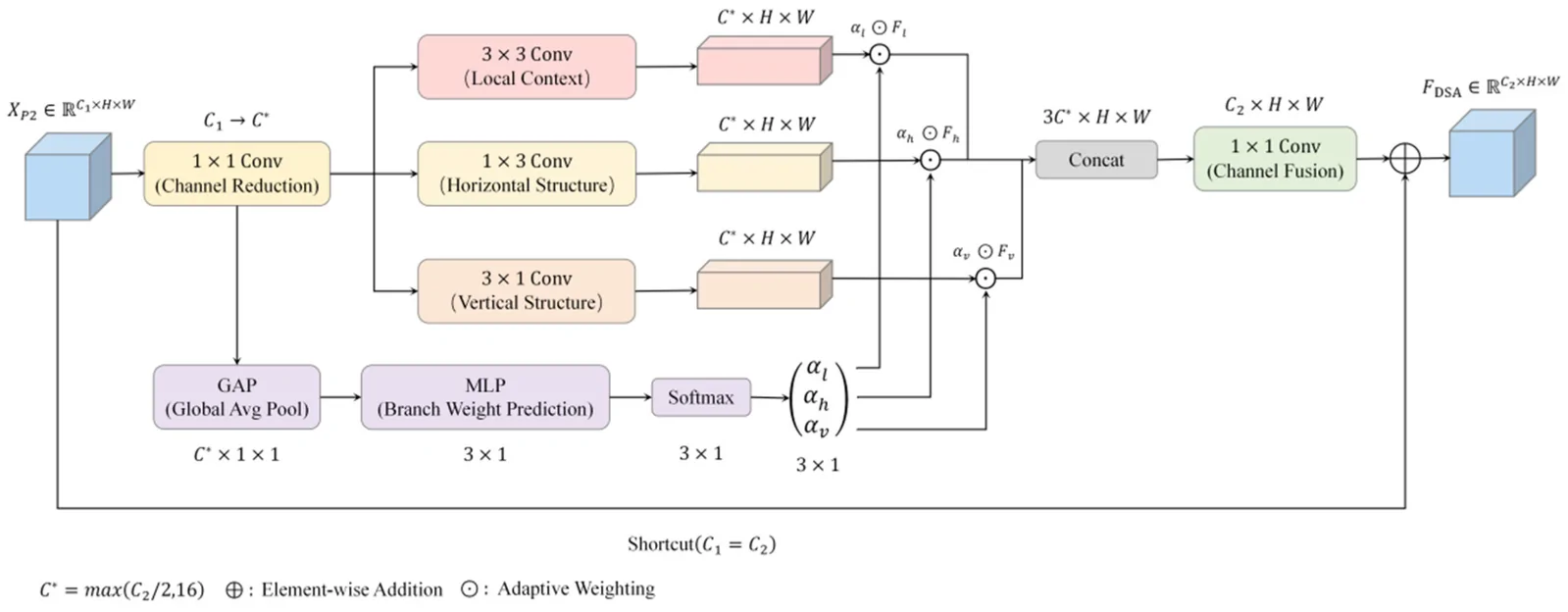
Architecture of the Directional Structure-Aware Module (DSA).

Pedestrian targets in UAV aerial imagery generally exhibit elongated shapes, while their short-side structures are easily compressed during network downsampling. For low-pixel pedestrians, this compression weakens the representation of head–shoulder contours, body boundaries, and local directional cues. Moreover, the relative importance of local, horizontal, and vertical structures varies with pedestrian pose, scale, and viewing angle. To account for these variations, a Directional Structure-Aware Module (DSA) is introduced at the high-resolution P2 feature level. DSA combines multidirectional convolutional modeling with input-dependent branch weighting, allowing the contributions of different structural cues to be adjusted according to the current feature state. The architecture of DSA is illustrated in Figure 5.

Given the high-resolution P2 input feature $X_{P2}\in R^{C_{1}\times H\times W}$, where $C_{1}$ denotes the number of input channels and H and W denote the height and width of the feature map, respectively, DSA first employs a 1×1 convolution to compress the channel dimension, producing an intermediate feature $X_{r}\in R^{C^{*}\times H\times W}$. Here, $C^{*}$ denotes the number of channels after compression. This operation reduces the channel dimensionality while preserving the spatial resolution, thereby decreasing the computational cost of the subsequent multi-branch convolutional modeling.

DSA then constructs three parallel directional structure modeling branches composed of 3×3, 1×3, and 3×1 convolutions. The 3×3 branch captures local two-dimensional neighborhood context to enhance the local structural representation of the target region. The 1×3 branch performs horizontal structure modeling, facilitating the extraction of transverse structural features such as the shoulders and head–shoulder connections of pedestrians. The 3×1 branch performs vertical structure modeling to strengthen directional information associated with torso boundaries and longitudinal contours. The feature extraction processes of the three branches can be formulated as

(4) $$F_{l}={Conv}_{3\times 3}\left(X_{r}\right),F_{h}={Conv}_{1\times 3}\left(X_{r}\right),\;F_{v}={Conv}_{3\times 1}\left(X_{r}\right).$$

where $F_{l}$, $F_{h}$, and $F_{v}$ denote the output features of the local, horizontal, and vertical branches, respectively. To overcome the limited adaptability of fixed multi-branch fusion to varying target structural states, adaptive weights for the three directional branches are further generated from the input features. Specifically, global average pooling is first applied to the intermediate feature $\left(X_{r}\right)$ to obtain a channel descriptor vector:

(5) $$z=GAP\left(X_{r}\right),$$

where $z\in R^{C^{*}\times 1\times 1}$. The global descriptor vector is then mapped through a multilayer perceptron (MLP) [25], and three normalized branch weights are generated using the Softmax function:

(6) $$\left[\alpha_{l},\alpha_{h},\alpha_{v}\right]=Softmax\left(MLP\left(z\right)\right),$$

$\alpha_{l}$, $\alpha_{h}$, and $\alpha_{v}$ denote the weights assigned to the local contextual, horizontal structural, and vertical structural branches, respectively, satisfying $\alpha_{l}+\alpha_{h}+\alpha_{v}=1$. Since the weight-generation branch operates only on the channel descriptor vector obtained through global average pooling, rather than directly processing the full spatial feature map, it introduces only a small number of additional parameters and limited computational overhead. After obtaining the branch weights, DSA adaptively reweights the outputs of the three directional branches as follows:

(7) $${\hat{F}}_{l}=\alpha_{l}⊙F_{l},\;{\hat{F}}_{h}=\alpha_{h}⊙F_{h},\;{\hat{F}}_{v}=\alpha_{v}⊙F_{v}.$$

Here, ⊙ denotes the adaptive weighting operation. Since $\alpha_{l}$, $\alpha_{h}$, and $\alpha_{v}$ are branch-level scalar weights, they are broadcast across the channel and spatial dimensions when multiplied by the corresponding branch features. This design does not select a single branch from the three alternatives. Instead, all three branches participate in feature extraction, while their relative contributions are dynamically adjusted according to the input features. Subsequently, the three weighted branch features are concatenated along the channel dimension:

(8) $$F_{cat}=Concat\left({\hat{F}}_{l},{\hat{F}}_{h},{\hat{F}}_{v}\right),$$

$Concat\left(\cdot \right)$ denotes concatenation along the channel dimension, and the resulting feature has a size of $3C^{*}\times H\times W$. To integrate structural information from different directions and restore the desired number of output channels, a 1×1 convolution is subsequently employed for channel fusion, producing the directionally enhanced feature:

(9) $$F_{dir}={Conv}_{1\times 1}\left(F_{cat}\right),$$

Finally, DSA employs a residual connection to fuse the directionally enhanced feature with the input feature:

(10) $$F_{DSA}=F_{dir}+Shortcut\left(X_{p2}\right),$$

where $F_{DSA}\in R^{C_{2}\times H\times W}$ denotes the output feature of the DSA module. When the numbers of channels in the input and output features are inconsistent, a 1×1 convolution can be introduced into the residual branch for channel alignment.

The DSA output integrates local, horizontal, and vertical features using input-dependent branch weights. Unlike fixed branch fusion, the relative contribution of each branch varies with the input feature state.

#### 2.2.2. Degradation-Guided Attention Module

Although the DSA Module enhances the local directional structural representation of small-scale pedestrians, directional convolutional modeling alone remains insufficient to adequately emphasize weak structural features in target regions that have already undergone or are close to undergoing short-side degradation. In UAV aerial scenes, background textures are often complex, and road edges, shadows, pole-like objects, as well as other small-scale distractors can produce feature responses similar to those of low-pixel pedestrians, making degradation-sensitive target regions difficult to distinguish effectively on the feature maps.

**Figure 6:**
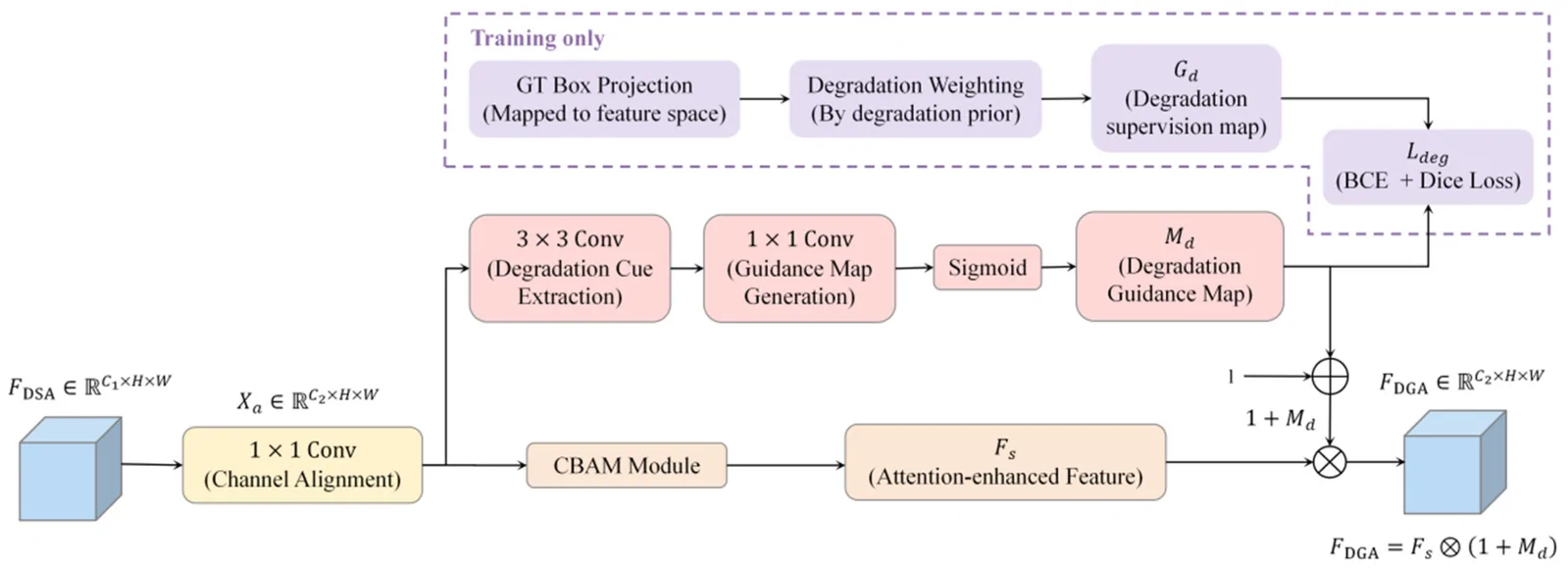
Architecture of the Degradation-Guided Attention Module (DGA).

To address this issue, a Degradation-Guided Attention Module (DGA) is designed. Specifically, DGA constructs a degradation-guided branch to predict a degradation guidance map, which is then used to spatially reweight degradation-sensitive regions, thereby improving the discriminability between small-scale pedestrians and complex backgrounds. Furthermore, to ensure that the learning of the degradation guidance map is consistent with the short-side structural degradation prior defined in this study, a degradation supervision map $G_{d}$ is introduced during training, and an auxiliary loss is employed to constrain the degradation guidance map $M_{d}$. The architecture of DGA is illustrated in Figure 6.

DGA plays a different role from the Area Attention (A2) mechanism in YOLOv12. A2 primarily provides general contextual modeling over feature regions, but does not explicitly identify or emphasize spatial regions affected by short-side structural degradation. In contrast, DGA is applied to the high-resolution P2 features and jointly performs channel-spatial refinement and degradation-guided reweighting under explicit degradation supervision. Therefore, A2 and DGA operate at different levels of feature modeling: A2 provides general contextual representations, whereas DGA selectively enhances degradation-sensitive local regions.

The DGA module takes the output feature of DSA, $F_{DSA}$, as its input. First, a 1×1 convolution is applied to align the channel dimension of the input feature, producing a channel-aligned feature $X_{a}\in R^{C_{2}\times H\times W}$, where $C_{2}$ denotes the number of aligned channels, and H and W denote the height and width of the feature map, respectively. This operation standardizes the feature channels while preserving the spatial resolution, thereby providing a consistent input representation for the subsequent degradation-guided and attention-enhancement branches.

In the degradation-guided branch, $X_{a}$ is sequentially processed by a 3×3 convolution and a 1×1 convolution. The 3×3 convolution extracts local spatial contextual information, whereas the 1×1 convolution compresses the channel dimension to produce a single-channel spatial weighting map. Subsequently, a Sigmoid activation function is applied to generate the degradation guidance map $M_{d}$:

(11) $$M_{d}=\sigma \left({Conv}_{1\times 1}\left({Conv}_{3\times 3}\left(X_{a}\right)\right)\right),$$

here, $\sigma \left(\cdot \right)$ denotes the Sigmoid function, and $M_{d}\in R^{1\times H\times W}$. The degradation guidance map characterizes the sensitivity of different spatial locations to structural degradation. A higher value indicates that the corresponding region is more likely to contain small-scale pedestrian features affected by short-side degradation, weakened boundaries, or incomplete local structures.

In the attention-enhancement branch, CBAM [26] is employed to adaptively enhance the channel-aligned feature $X_{a}$. CBAM consists of two sequential stages: channel attention and spatial attention. First, the channel attention module generates the channel attention weights $M_{C}\left(X_{a}\right)$ and recalibrates $X_{a}$ along the channel dimension, producing the channel-attention-enhanced feature $F_{c}$. Subsequently, the spatial attention module generates the spatial attention weights $M_{s}\left(F_{c}\right)$ from $F_{c}$ and further recalibrates the importance of the feature responses at different spatial locations, yielding the attention-enhanced feature $F_{s}$:

(12) $$F_{c}=M_{C}\left(X_{a}\right)⊗X_{a},F_{s}=M_{s}\left(F_{c}\right)⊗F_{c}.$$

$M_{C}\left(\cdot \right)$ and $M_{s}\left(\cdot \right)$ denote the channel attention map and spatial attention map in CBAM, respectively. $F_{c}$ represents the intermediate feature enhanced by channel attention, whereas $F_{s}$ denotes the attention-enhanced feature obtained after sequential channel and spatial attention refinement. The symbol ⊗ denotes element-wise multiplication. Through adaptive recalibration along both the channel and spatial dimensions, the attention-enhancement branch emphasizes informative channels and critical spatial locations associated with small-scale pedestrians, thereby providing more discriminative base features for enhancing degradation-sensitive regions. Finally, DGA employs the degradation guidance map $M_{d}$ to perform residual-style reweighting of the attention-enhanced feature $F_{s}$. Specifically, $1+M_{d}$ is used as the spatial enhancement weight and element-wise multiplied by $F_{s}$, yielding the output feature of the Degradation-Guided Attention Module, $F_{DGA}:$

(13) $$F_{DGA}=F_{s}⊗\left(1+M_{d}\right),$$

where $F_{DGA}$ denotes the output feature of the DGA module. Since $1+M_{d}$ is a single-channel spatial guidance map, it is broadcast along the channel dimension when element-wise multiplied by the multi-channel feature $F_{s}$, thereby enabling spatial reweighting across all feature channels. Compared with directly weighting the feature using $M_{d}$, the residual-style reweighting scheme based on ${1+M}_{d}$ further enhances degradation-sensitive regions while preserving the original attention-enhanced feature. This design prevents weak small-scale pedestrian features from being excessively suppressed due to insufficient degradation-guidance weights.

To align the learning objective of $M_{d}$ with the short-side degradation prior, a degradation supervision map $G_{d}$ is introduced during training to explicitly constrain $M_{d}$ through an auxiliary loss. Since $M_{d}$ is generated by the degradation-guided branch and has a spatial size of 1×H×W, $G_{d}$ is constructed as a single-channel supervision map of the same size. First, the ground-truth bounding boxes in the input image are projected onto an H×W supervision grid according to the downsampling stride s of the current feature level, establishing a spatial correspondence between the ground-truth target regions and the locations in $M_{d}$. For the i-th ground-truth pedestrian bounding box, let $w_{i}$ and $h_{i}$ denote its width and height in the input image, respectively. Its mapped short-side dimension at the current feature level is then calculated as

(14) $$d_{i}=\frac{min\left(w_{i},h_{i}\right)}{s},$$

where $d_{i}$ denotes the mapped short-side dimension of the i-th target at the feature level where DGA is applied. According to the definition of the degradation level, when $d_{i}\leq 1$, the short-side extent of the target has been reduced to no more than one feature unit through successive scale mappings. After discrete sampling on the feature map, its local contours, boundaries, and directional structures can no longer be jointly represented by multiple adjacent feature units, indicating a state of substantial structural degradation. Furthermore, when the mapped short-side dimension spans approximately two feature units, the target can still retain a basic multi-unit spatial representation. Based on this consideration, an upper degradation threshold τ is introduced to define the near-degradation range, with *τ* = 2 adopted as the default setting in this study. Accordingly, a degradation weight is assigned to each ground-truth pedestrian bounding box based on its mapped short-side dimension:

(15) $$\rho_{i}=\left\{\begin{array}{cc} 1, & d_{i}\leq 1,\\ \frac{\tau -d_{i}}{\tau -1}, & 1<d_{i}<\tau ,\\ 0, & d_{i}\geq \tau , \end{array}\right.\;\tau >1$$

$\rho_{i}\in [0,1]$ denotes the degradation degree of the i-th target. When $d_{i}\leq 1$, the target exhibits a high degree of structural degradation, and its degradation weight is set to 1. When $1<d_{i}<2$, the target is considered to be in a near-degradation state, and the degradation weight decreases as the mapped short-side dimension increases. When $d_{i}\geq 2$, the short-side structure of the target can still retain basic spatial information, and the degradation weight is set to 0.

Subsequently, the degradation supervision map $G_{d}$ is generated according to the degradation weight $\rho_{i}$. Let $B_{i}^{s}$ denote the region obtained by projecting the i-th ground-truth bounding box onto the H×W grid according to the downsampling stride s of the current feature level. Then, $G_{d}$ can be expressed as

(16) $$G_{d}\left(x,y\right)=\underset{i}{\max}\left(\rho_{i}\cdot 1_{(x,y)\in B_{i}^{s}}\right),$$

$1_{(x,y)\in B_{i}^{s}}$ denotes the indicator function, which takes a value of 1 when the spatial location $\left(x,y\right)$ lies within the projected target region $B_{i}^{s}$, and 0 otherwise. When multiple projected target regions overlap, the maximum degradation weight associated with that location is assigned as the supervision value. In this manner, pedestrian regions exhibiting more severe short-side compression are assigned higher supervision weights in $G_{d}$, thereby providing a clearer learning objective for the degradation guidance map $M_{d}$.

During training, the degradation supervision map $G_{d}$ is used to provide auxiliary supervision for the degradation guidance map $M_{d}$ predicted by the degradation-guided branch. The degradation auxiliary loss is jointly constructed using the binary cross-entropy (BCE) loss [27] and Dice loss [28]:

(17) $$L_{deg}=L_{BCE}\left(M_{d},G_{d}\right)+L_{Dice}\left(M_{d},G_{d}\right),$$

the BCE loss constrains the predicted degradation values at individual spatial locations, whereas the Dice loss improves the overlap consistency between the predicted degradation-sensitive regions and the corresponding supervision regions. In the implementation, $L_{BCE}$ and $L_{Dice}$ are combined with equal weights, and no additional internal balancing coefficients are introduced. The overall training loss of the model is finally formulated as

(18) $$L_{total}=L_{det}+\lambda_{deg}L_{deg},$$

where $L_{det}$ denotes the original detection loss of the YOLO detection head, and $\lambda_{deg}$ is the weighting coefficient of the degradation auxiliary loss. In the subsequent experiments, the same $\lambda_{deg}$ setting is adopted for all models. During inference, the degradation supervision map and auxiliary loss are removed, while the forward computation of DGA is retained. The predicted degradation guidance map is used to spatially reweight the attention-enhanced feature. Therefore, the degradation prior introduces no supervision-related computation during inference.

#### 2.2.3. Fine-Grained Structure Preservation Module

**Figure 7:**
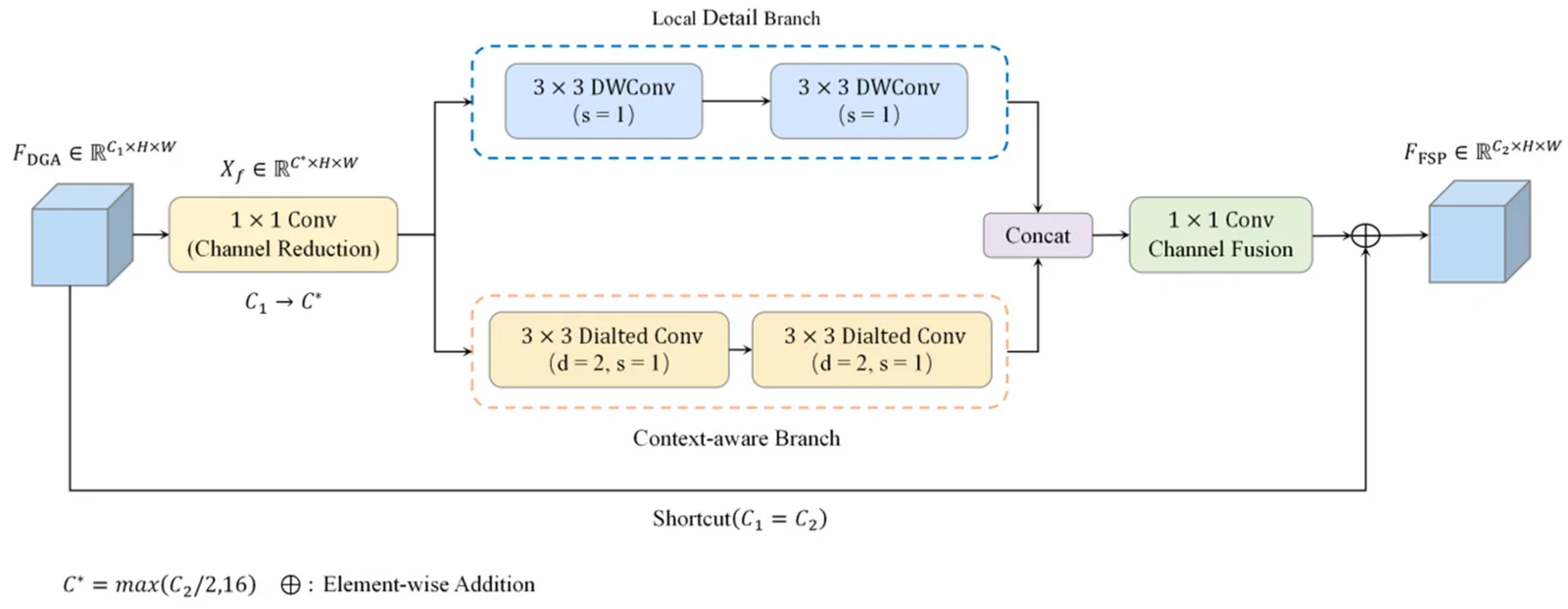
Architecture of the Fine-Grained Structure Preservation Module (FSP).

After DSA and DGA enhance the directional structures and degradation-sensitive regions of small-scale pedestrians, local contours and neighborhood contextual information may still be weakened during subsequent feature transformation. To preserve these complementary cues, a Fine-Grained Structure Preservation Module (FSP) is introduced. FSP combines local-detail modeling with neighborhood-context modeling to retain target boundaries and improve the distinction between low-pixel pedestrians and surrounding background structures. The architecture of FSP is illustrated in Figure 7.

The FSP module takes the output feature of the Degradation-Guided Attention Module, $F_{DGA}$, as its input. First, a 1×1 convolution is employed to compress the channel dimension of the input feature, producing an intermediate feature $X_{f}\in R^{C^{*}\times H\times W}$, where $C^{*}$ denotes the number of intermediate channels after compression. Subsequently, FSP constructs a local-detail branch and a context-aware branch to preserve the local structural details and neighborhood contextual information of the target, respectively. In the local-detail branch, $X_{f}$ is sequentially processed by two 3×3 depthwise convolutions (DWConv) [29] with a stride of 1. These depthwise convolutions are used to enhance fine-grained structural features, including target boundaries, contours, and local textures. This process can be formulated as

(19) $$F_{local}={DWConv}_{3\times 3}^{d=1,s=1}\left({DWConv}_{3\times 3}^{d=1,s=1}\left(X_{f}\right)\right),$$

where $F_{local}$ denotes the output feature of the local-detail branch. Compared with standard convolution, depthwise convolution enables local spatial modeling with lower computational overhead, making it well suited for preserving the fine-grained structural information of small-scale pedestrians at the high-resolution P2 feature level.

In the context-aware branch, $X_{f}$ is sequentially processed by two 3×3 dilated convolutions [30], each with a dilation rate of 2 and a stride of 1. These convolutions enlarge the receptive field without reducing the spatial resolution of the feature map, thereby enhancing the representation of contextual information in the target neighborhood. This process can be formulated as

(20) $$F_{context}={Dilated\;Conv}_{3\times 3}^{d=2,s=1}\left({Dilated\;Conv}_{3\times 3}^{d=2,s=1}\left(X_{f}\right)\right),$$

where $F_{context}$ denotes the output feature of the context-aware branch, and d denotes the dilation rate. By introducing dilated convolutions, this branch captures spatial contextual information over a larger range, thereby enhancing the structural differences between small-scale pedestrians and the surrounding background. Subsequently, the output features of the local-detail and context-aware branches are concatenated along the channel dimension to obtain the fused feature $F_{cat}$:

(21) $$F_{cat}=Concat\left(F_{local},F_{context}\right),$$

$Concat\left(\cdot \right)$ denotes concatenation along the channel dimension. Since both branches produce $C^{*}$ output channels, the concatenated feature contains ${2C}^{*}$ channels. To integrate the local-detail and contextual structural information, FSP further employs a 1×1 convolution for channel fusion, yielding the fine-grained structure fusion feature $F_{fg}$:

(22) $$F_{fg}={Conv}_{1\times 1}\left(F_{cat}\right),$$

Finally, FSP employs a residual connection to add the fine-grained structure fusion feature to the input feature, producing the output feature of the module, $F_{FSP}$:

(23) $$F_{FSP}=F_{fg}+F_{DGA}.$$

where $F_{FSP}$ denotes the output feature of the Fine-Grained Structure Preservation Module. The residual connection preserves the original semantic and spatial information contained in the input feature, thereby preventing the loss of informative target features during multi-branch convolutional modeling while improving the stability of network training.

## 3. Experiments

### 3.1. Experimental Data and Environment

The experiments in this study were conducted on the VisDrone2019 object detection dataset (VisDrone2019-DET). The training, validation, and annotated test-dev subsets contain 6471, 548, and 1610 images, respectively. Model parameters were optimized on the training set, and the best-performing weights were selected according to the detection performance on the validation set. Subsequently, the test-dev subset with ground-truth annotations was used as the test set for the quantitative evaluation of model predictions.

VisDrone2019-DET contains ten object categories. For the single-class pedestrian detection task considered in this study, annotations belonging to categories other than “pedestrian” and “people” were removed, while the “pedestrian” and “people” categories were merged into a single pedestrian category. Objects from the remaining categories were retained in the original images but were not treated as positive samples during training. During testing, the detection metrics were calculated exclusively using the merged pedestrian annotations. All models were compared under identical data preprocessing and evaluation settings.

All experiments were conducted on a local laptop running Windows 11 and equipped with an NVIDIA GeForce RTX 4070 GPU. The software environment consisted of Python 3.10.18, PyTorch 2.7.1, and CUDA 11.8. All models were implemented using the Ultralytics-based YOLOv12 framework and evaluated under identical hyperparameter settings. The input image resolution was set to 640 × 640, the batch size was set to 4, the initial learning rate was set to 0.01, and the models were trained for 300 epochs.

The batch size was set to 4 primarily due to the GPU memory limitation of the experimental platform, particularly because the introduced high-resolution P2 feature substantially increases memory consumption during training. A relatively small batch size may increase the stochasticity of gradient estimates and Batch Normalization statistics, and may therefore affect the absolute convergence behavior and generalization performance of the model. Nevertheless, no evident training instability was observed in our experiments. Moreover, all compared models were trained using the same batch size and hyperparameter settings, ensuring a controlled and consistent basis for relative performance comparison. The detailed training settings for VisDrone2019-DET are presented in Table 1.

### 3.2. Evaluation Metrics

The proposed method is comprehensively evaluated in terms of model complexity, inference speed, and detection accuracy. Model complexity is measured using the number of parameters (Params) and floating-point operations (GFLOPs), while inference speed is quantified by frames per second (FPS). Detection accuracy is evaluated using mean Average Precision (mAP), Precision (P), and Recall (R). The definitions of these evaluation metrics are provided in Equations (24) and (25).

(24) $$P=\frac{TP}{TP+FP},\;R=\frac{TP}{TP+FN}.$$

(25) $$AP=\int_{0}^{1}PdR,\;mAP=\frac{1}{N}\sum_{i=1}^{N}AP_{i}.$$

*P* denotes the proportion of correctly predicted positive samples among all samples predicted as positive, whereas *R* denotes the proportion of actual positive samples successfully detected by the model. True positives (*TP*) refer to positive samples correctly detected by the model, false positives (*FP*) refer to negative samples incorrectly predicted as positive, and false negatives (*FN*) refer to positive samples missed by the model. The *mAP* is defined as the macro-average of the average precision (*AP*) values over *N* categories and is used to comprehensively evaluate the overall detection performance of the model. For each category, *AP* is calculated as the area under the Precision–Recall (P–R) curve. Since the evaluation involves a single merged pedestrian category, N=1, and the reported *mAP* is numerically equivalent to the *AP* of the pedestrian class.

### 3.3. Ablation Studies and Effectiveness Analysis

#### 3.3.1. Analysis of Ablation Study Results

To evaluate the effectiveness of each proposed component, ablation experiments were conducted using YOLOv12n as the baseline model. The high-resolution P2 detection layer, DSA, DGA, and FSP were progressively incorporated into the baseline. P, R, mAP50, mAP50-95, the number of parameters, computational complexity, and inference speed were adopted as the evaluation metrics. The experimental results are presented in Table 2, where A, B, C, and D denote the P2 detection layer, DSA, DGA, and FSP, respectively. For experiments involving DGA, the weighting coefficient of the degradation auxiliary loss, $\lambda_{deg}$ was set to 0.1 by default.

As shown in Table 2, adding the P2 detection layer increases recall from 44.4% to 49.7% and mAP50 from 49.4% to 56.4%, representing the largest performance gain among the evaluated components. This result suggests that introducing a higher-resolution detection level is the main source of improvement for the present task.

On the P2 configuration, DSA further increases mAP50 and mAP50-95 to 58.1% and 25.6%, respectively. DGA and FSP provide additional gains in the combined configurations, and the complete model reaches 70.4% precision, 51.8% recall, 59.7% mAP50, and 26.9% mAP50-95.

These improvements are accompanied by an increase in computation from 6.5 to 18.8 GFLOPs and a decrease in inference speed from 35.9 to 15.7 FPS. DGF-YOLO therefore provides higher detection accuracy and recall at the cost of increased computation on high-resolution features.

**Figure 8:**
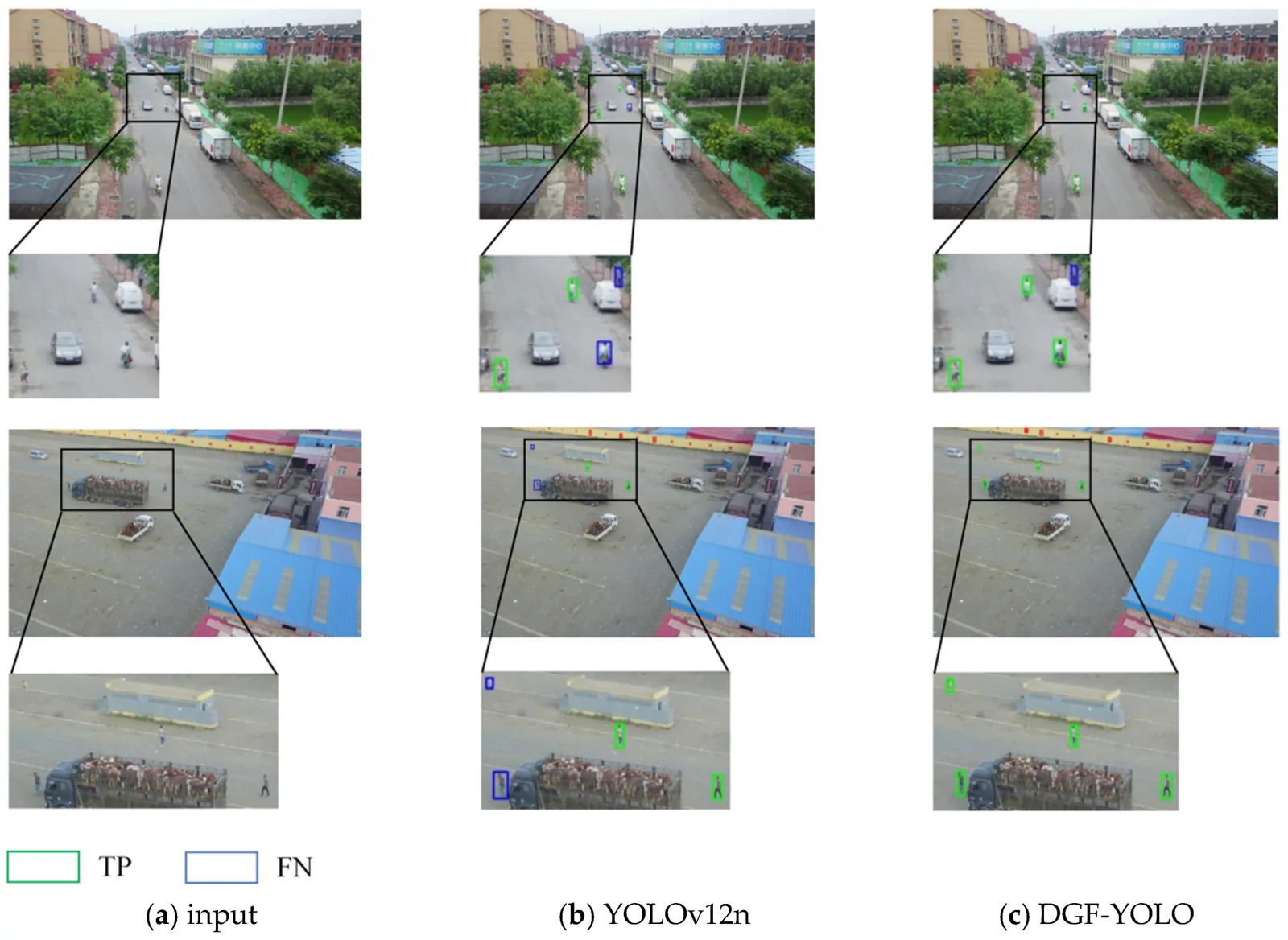
Qualitative comparison of detection results between YOLOv12n (**b**) and DGF-YOLO (**c**). (**a**) is the input. The black boxes indicate the regions selected for enlarged visualization.

Figure 8 presents the test results of YOLOv12n and DGF-YOLO. The green boxes indicate correctly detected pedestrians, whereas the blue boxes denote missed detections. As shown in the figure, DGF-YOLO detects more pedestrian targets than the YOLOv12n baseline, thereby reducing the number of missed detections.

**Figure 9:**
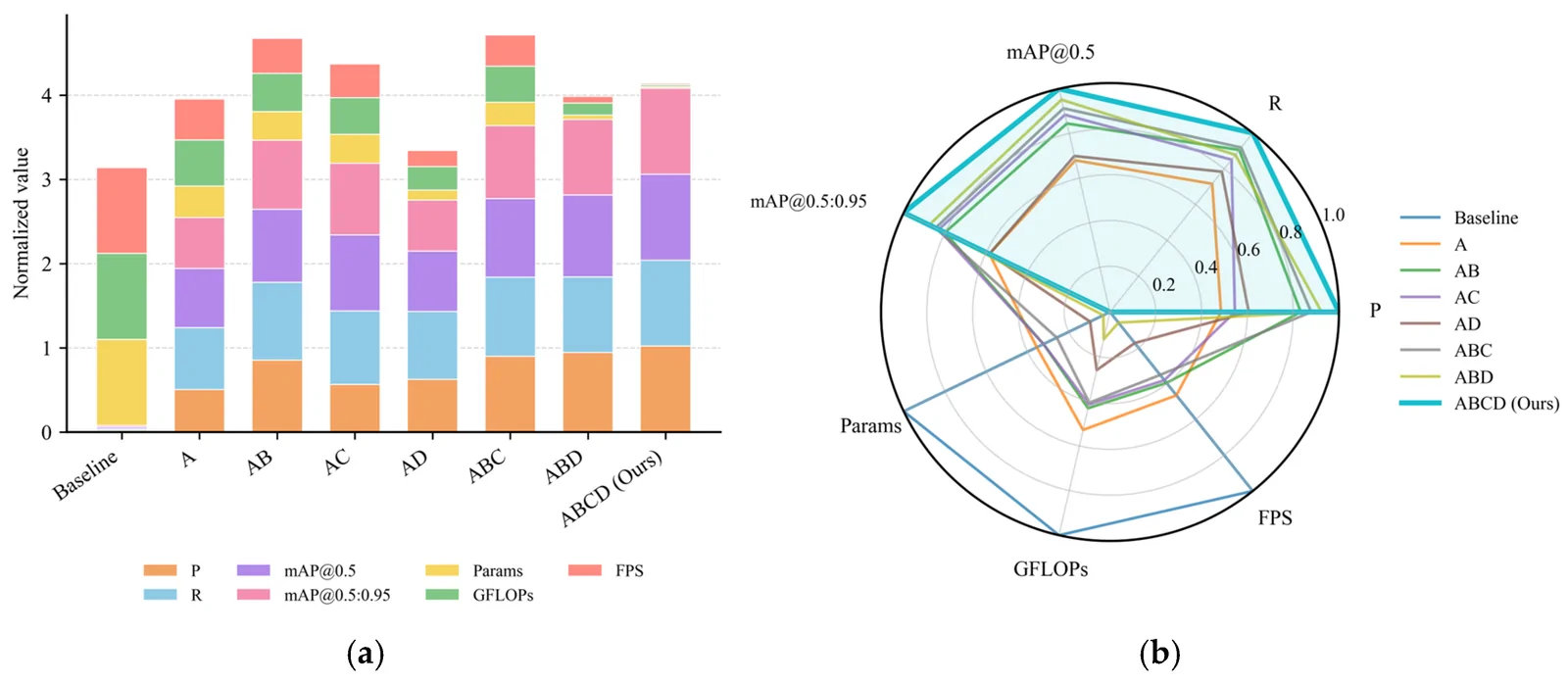
Normalized performance of the ablation experiments on the VisDrone2019-DET dataset: (**a**) normalized bar chart; (**b**) performance radar chart.

To further provide an intuitive comparison of the overall performance of different ablation models in terms of detection accuracy, model complexity, and inference speed, all the metrics listed in the table were normalized, and the corresponding normalized bar chart and radar chart were plotted, as shown in Figure 9.

Figure 9 provides a visual summary of the accuracy–efficiency trade-off already reported in Table 2.

#### 3.3.2. Sensitivity Analysis of the Degradation Auxiliary Loss Weight

To investigate the effect of the degradation auxiliary loss weight on model performance, sensitivity experiments were conducted on the P2 + DGA model using different values of $\lambda_{deg}$. Except for $\lambda_{deg}$, all network structures, training parameters, and testing settings were kept identical. When $\lambda_{deg}=0$, only the forward degradation-guided enhancement process of the DGA module was retained, while the degradation auxiliary loss $L_{deg}$ was disabled. The experimental results are presented in Table 3.

As shown in Table 3, when $\lambda_{deg}=0$, the model retains only the forward degradation-guided enhancement process of DGA without using the degradation auxiliary loss. In this case, $M_{d}$ is learned indirectly through the detection loss, resulting in only limited performance improvement. As $\lambda_{deg}$ increases from 0 to 0.1, the supervision imposed by the degradation supervision map $G_{d}$ becomes progressively stronger, accompanied by consistent improvements in detection performance. However, when $\lambda_{deg}$ is further increased to 0.15 and 0.2, P improves slightly, whereas R and mAP decrease. This suggests that an excessively large auxiliary-loss weight may cause the model to overemphasize degradation-region supervision, thereby interfering with the optimization of the primary detection task. Considering detection accuracy, recall, and localization performance comprehensively, $\lambda_{deg}$ is set to 0.1 in this study.

#### 3.3.3. Sensitivity Analysis of the Degradation Threshold

To evaluate the effect of the degradation threshold τ on DGA, sensitivity experiments were conducted on the P2 + DGA model with τ set to 1.5, 2.0, 2.5, and 3.0, while all other settings were kept unchanged. The experimental results are presented in Table 4. The model achieves the best overall performance at τ=2.0, with P, R, mAP50, and mAP50-95 reaching 67.4%, 50.7%, 58.5%, and 25.8%, respectively. When τ is reduced to 1.5, only minor performance variations are observed, indicating that the model is not highly sensitive to moderate changes in the threshold. In contrast, increasing τ to 2.5 and 3.0 leads to a gradual decline in detection performance because an excessively large threshold expands the degradation-supervised range to targets that still retain relatively sufficient spatial representations. Therefore, τ=2.0 provides an appropriate balance between degradation sensitivity and supervision specificity and is adopted in this study.

#### 3.3.4. Ablation Study on the Internal Structure of the DSA Module

To evaluate the effectiveness of the different convolutional branches and the adaptive branch-weighting mechanism within the DSA Module, the model equipped with the high-resolution P2 detection layer was adopted as the baseline. Ablation variants incorporating the local convolution branch, directional convolution branches, multi-branch convolution combinations, and the complete DSA module were constructed and evaluated separately. Except for the DSA-related structural configurations, all training parameters and testing settings were kept identical. The experimental results are presented in Table 5.

As shown in Table 5, the local branch alone provides only a small improvement over P2. Larger gains are obtained after the horizontal and vertical branches are introduced, while combining all three branches further improves mAP50 and mAP50-95 to 57.7% and 24.9%, respectively.

Compared with the fixed three-branch configuration, the complete DSA module further improves P, R, mAP50, and mAP50-95 by 1.0, 0.6, 0.4, and 0.7 percentage points, respectively. Meanwhile, the adaptive weighting mechanism increases the number of parameters only from 2.709 M to 2.710 M and the computational cost from 13.2 to 13.5 GFLOPs. This limited additional computational cost is mainly due to the weight-generation branch operating on the compact channel descriptor obtained through global average pooling rather than on the full spatial feature map. These results indicate that adaptive branch weighting provides a favorable trade-off between detection performance and computational complexity.

#### 3.3.5. Comparative Experiments on Different Attention Mechanisms

To compare DGA with generic attention mechanisms, representative modules, including ECA [31], CA [32], EMA [33], and CBAM, were selected for comparative experiments using the model equipped only with the high-resolution P2 detection layer as the baseline. All attention modules were inserted at the same P2 feature-enhancement position. Except for the attention mechanism employed, the remaining network architecture, training parameters, and testing settings were kept identical. The experimental results are presented in Table 6.

Under the same insertion position and training settings, ECA, CA, and EMA do not outperform the P2 baseline. CBAM provides modest improvements in recall, mAP50, and mAP50-95, whereas DGA achieves the highest values among the evaluated attention modules.

Compared with P2, DGA improves precision, recall, mAP50, and mAP50-95 by 0.4, 1.0, 2.1, and 1.6 percentage points, respectively. These results suggest that degradation-guided supervision is beneficial for the present task configuration.

Since the P2 baseline retains the native A2 mechanism of YOLOv12, the additional performance gains obtained by DGA indicate that degradation-guided refinement provides complementary information rather than merely duplicating the feature recalibration performed by A2. Moreover, the smaller improvement obtained by directly adding CBAM compared with DGA indicates that the gain of DGA does not arise solely from stacking another generic attention mechanism, but is mainly associated with the degradation-guided branch and its explicit supervision.

#### 3.3.6. Ablation Study on the Internal Structure of the FSP Module

To further investigate the roles of the different convolutional branches within the FSP module, three variants, namely P2 + DWConv, P2 + Dilated Conv, and P2 + FSP, were constructed and comparatively evaluated using the model equipped only with the high-resolution P2 detection layer as the baseline. The experimental results are presented in Table 7.

As shown in Table 7, neither DWConv nor dilated convolution alone outperforms the P2 baseline. The complete FSP configuration increases precision from 67.0% to 67.8%, recall from 49.7% to 50.2%, and mAP50 from 56.4% to 56.6%, while mAP50-95 remains unchanged at 24.2%.

The combined configuration performs better than either single-branch variant, suggesting that local and contextual branches are more suitable when used jointly. However, the gain over the P2 baseline is modest.

#### 3.3.7. Detection Performance Analysis for Targets at Different Degradation Levels

This section analyzes the detection capability of different models for pedestrian targets with varying degrees of structural degradation, thereby further verifying the effectiveness of the proposed method in reducing missed detections of degraded small-scale pedestrians. According to the feature level at which each target first undergoes structural degradation during network downsampling, pedestrian targets in the test set are divided into four categories: P2-deg, P3-deg, P4-deg, and P5-deg. The recall of different models for targets at each degradation level is then calculated, and the results are presented in Table 8. Specifically, P2-deg, P3-deg, P4-deg, and P5-deg denote targets whose initial degradation levels are P2, P3, P4, and P5, respectively. All values reported for these categories represent the corresponding recall rates in percentage terms, while Overall denotes the recall rate calculated over all pedestrian targets.

As shown in Table 8, the recall of YOLOv12n increases from 39.8% for P2-deg targets to 63.3% for P5-deg targets. This trend indicates that targets assigned to earlier degradation levels are more difficult to detect under the present evaluation setting.

Adding P2 improves recall for all degradation-level groups, with the largest gains observed for P3-deg and P4-deg targets. The complete DGF-YOLO model provides further gains of 2.5, 1.8, 2.3, and 0.4 percentage points over P2 for the P2-deg, P3-deg, P4-deg, and P5-deg groups, respectively. The additional improvement is more evident for P2-deg to P4-deg targets, whereas the gain for P5-deg targets is limited.

#### 3.3.8. Visualization Analysis of the Degradation Guidance Map

**Figure 10:**
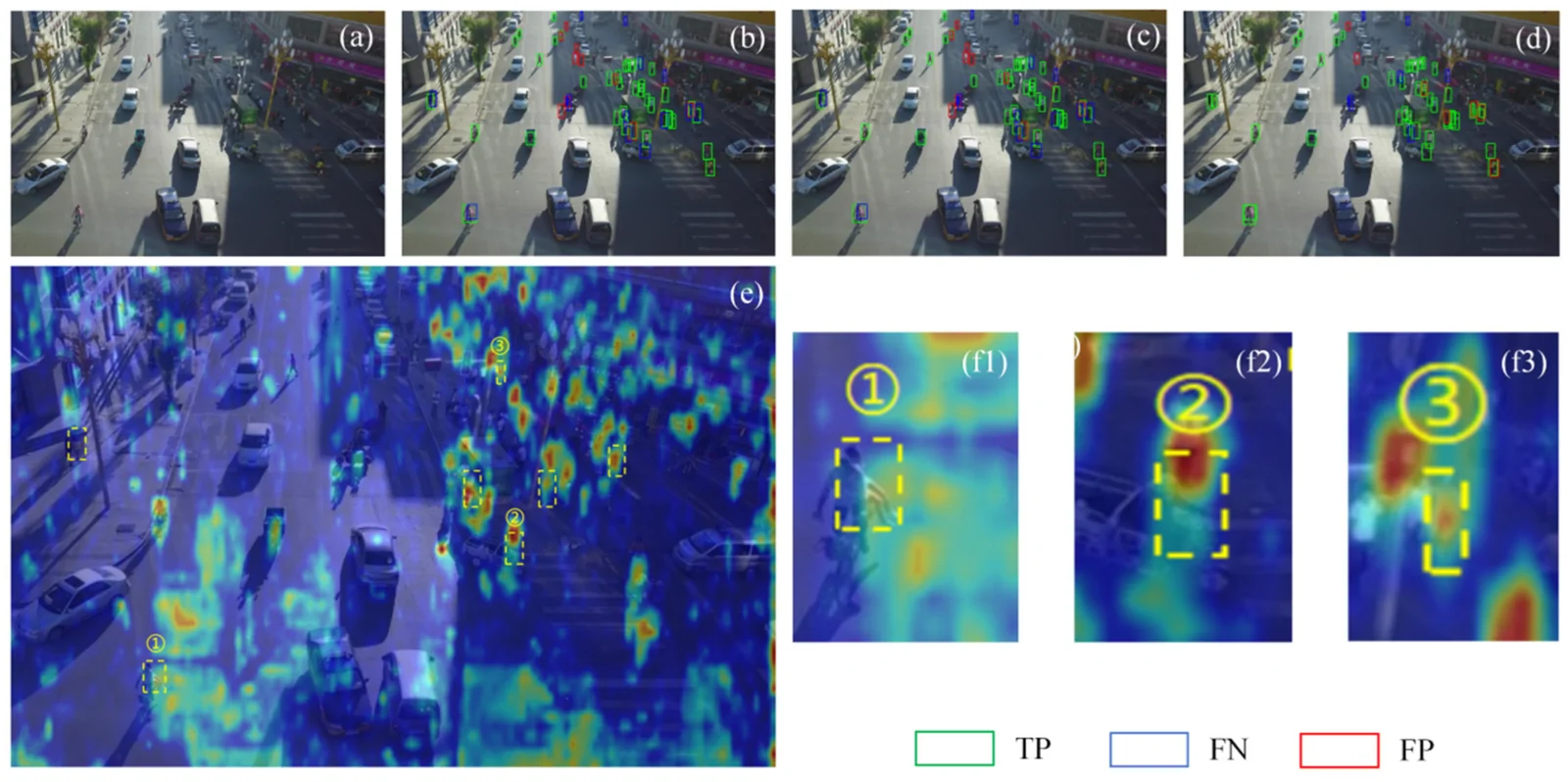
Visualization results of the degradation guidance maps. (**a**) Original image; (**b**) ground-truth annotations; (**c**) detection results of YOLOv12n; (**d**) detection results of DGF-YOLO; (**e**) degradation guidance map generated by the DGA module; and (**f1**–**f3**) enlarged views of representative degraded regions. The yellow dashed boxes indicate small-scale pedestrian regions that were missed by YOLOv12n but successfully detected by DGF-YOLO. In the degradation guidance map, red and yellow regions indicate higher degradation-guidance weights, whereas blue regions indicate lower degradation-guidance weights.

To analyze the enhancement mechanism of the DGA module in regions containing degraded small-scale pedestrians, the degradation guidance maps generated by the degradation-guided branch were visualized and analyzed, as shown in Figure 10.

Compared with YOLOv12n, DGF-YOLO detects more pedestrians in the highlighted regions. The corresponding areas generally exhibit relatively high weights in the degradation guidance map, including several targets missed by the baseline model. This spatial correspondence suggests that DGA assigns increased weights to some low-pixel pedestrian regions and their local neighborhoods.

The guidance map should not be interpreted as a pedestrian segmentation result. Responses are also observed around vehicle edges, shadows, and road textures, indicating that the map captures spatially salient or degradation-sensitive structures rather than pedestrian locations alone.

### 3.4. Comparative Experiments

To validate the effectiveness of the proposed DGF-YOLO for small-scale pedestrian detection in UAV aerial imagery, it was compared with several mainstream object detection algorithms. In addition, YOLOv12s and YOLOv12m were included to examine whether the performance gains of DGF-YOLO are mainly attributable to increased model capacity and computational cost. The experimental results are presented in Table 9.

YOLOX-Nano and YOLOv5n achieve relatively high inference speeds but lower detection accuracy than the other evaluated models. YOLOv8n, YOLOv11n, and YOLOv12n exhibit comparable performance, with mAP50 values ranging from 48.6% to 49.4%. TPH-YOLOv5n improves upon YOLOv5n, while sliced training increases the mAP50 of YOLOv12n from 49.4% to 50.6%, with only a marginal change in recall from 44.4% to 44.5%. Increasing the model scale from YOLOv12n to YOLOv12s and YOLOv12m progressively improves detection performance, with mAP50 increasing to 50.7% and 53.5%, respectively. However, these gains are accompanied by a substantial increase in model complexity. Notably, YOLOv12s requires 19.6 GFLOPs, which is comparable to the 18.8 GFLOPs of DGF-YOLO. Under a similar computational budget, DGF-YOLO improves precision, recall, mAP50, and mAP50-95 by 5.2, 7.0, 9.0, and 5.1 percentage points, respectively, while using only 2.8 M parameters compared with 6.9 M for YOLOv12s. Furthermore, although YOLOv12m requires 17.8 M parameters and 47.3 GFLOPs, DGF-YOLO still outperforms it by 6.2 and 2.8 percentage points in mAP50 and mAP50-95, respectively.

DGF-YOLO achieves the highest precision, recall, mAP50, and mAP50-95 among all evaluated methods, reaching 70.4%, 51.8%, 59.7%, and 26.9%, respectively. Although the additional P2 detection layer and feature enhancement modules introduce extra computational overhead and reduce inference speed, DGF-YOLO still maintains substantially lower parameter count and computational cost than YOLOv12m while achieving superior detection accuracy.

**Figure 11:**
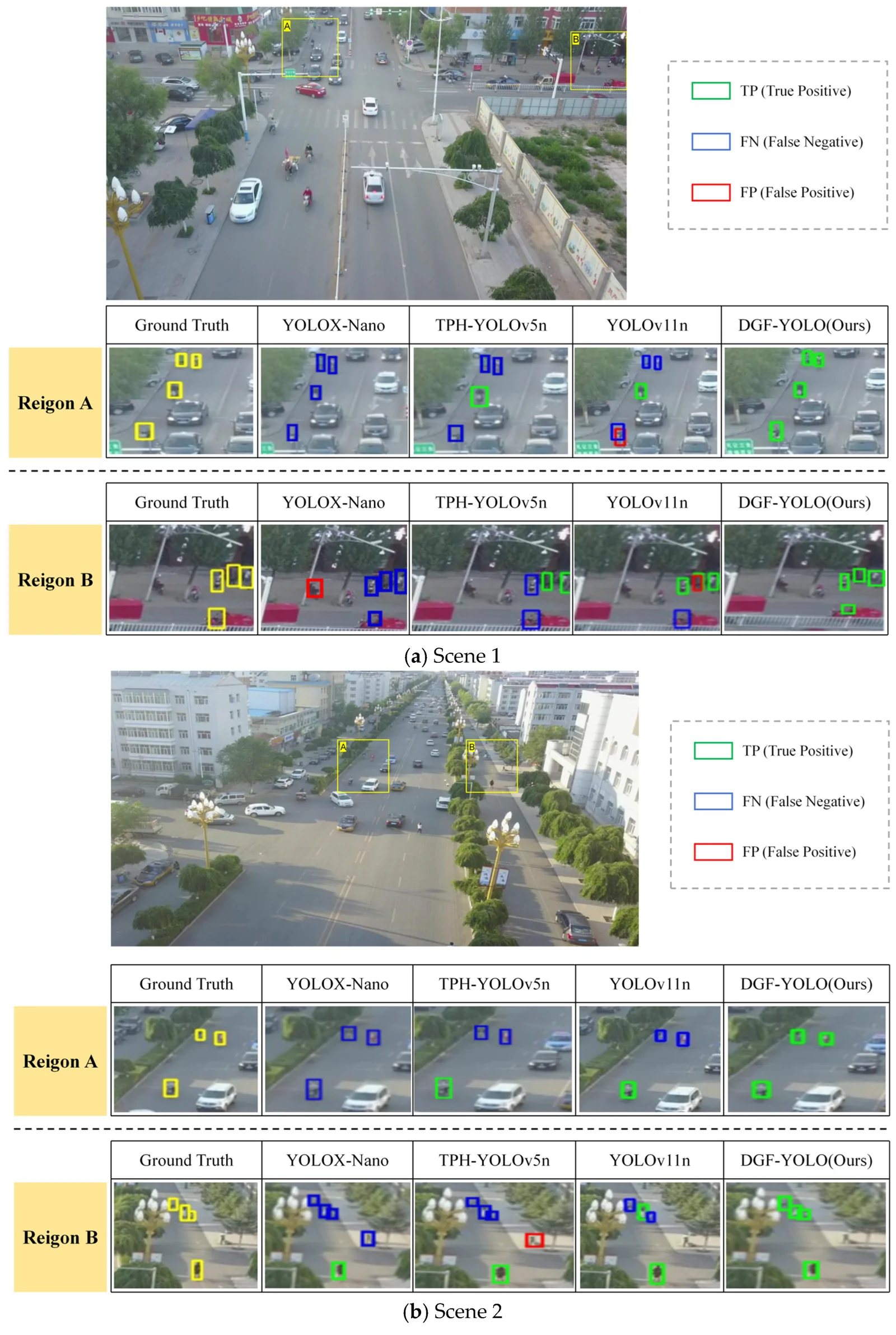
Qualitative comparison of different methods in challenging regions of the VisDrone2019-DET dataset. (**a**) Scene 1; (**b**) Scene 2. Green, blue, and red boxes denote true positives (TP), false negatives (FN), and false positives (FP), respectively. Yellow boxes indicate the ground-truth bounding boxes and the regions selected for enlarged visualization.

To further validate the practical detection performance of DGF-YOLO in complex aerial scenes, two representative test examples were selected for visual comparison, as shown in Figure 11.

It can be observed from the figure that YOLOX-Nano produces more missed detections in distant and low-pixel pedestrian regions. TPH-YOLOv5n and YOLOv11n detect more targets but still miss several pedestrians in densely distributed or visually ambiguous areas. Among the displayed results, DGF-YOLO provides the most complete target coverage.

### 3.5. Generalization Experiments on the Full VisDrone2019-DET Dataset

To evaluate the cross-category generalization of DGF-YOLO, additional experiments were conducted on the full VisDrone2019-DET dataset containing all ten object categories. Unlike the primary single-class pedestrian experiments, the original category annotations were retained without merging or filtering. The dataset split and training settings remained consistent with those described in Section 3.1, including an input size of 640 × 640 and 300 training epochs. No category-specific architectural modifications or parameter tuning were introduced. For DGA, the degradation supervision map was generated from the short-side dimensions of ground-truth boxes for all categories using the same degradation criterion as in the pedestrian experiments. As shown in Table 10, DGF-YOLO achieves 51.7% P, 40.4% R, 43.8% mAP50, and 22.3% mAP50-95, outperforming the YOLOv12n baseline by 6.8, 7.0, 9.1, and 4.8 percentage points, respectively. It also surpasses the larger YOLOv12s and YOLOv12m models in both mAP50 and mAP50-95 while maintaining lower model complexity. These results indicate that the proposed degradation-guided feature enhancement strategy remains effective across multiple object categories within VisDrone2019-DET, demonstrating its applicability beyond the single-class pedestrian detection setting.

## 4. Discussion

**Figure 12:**
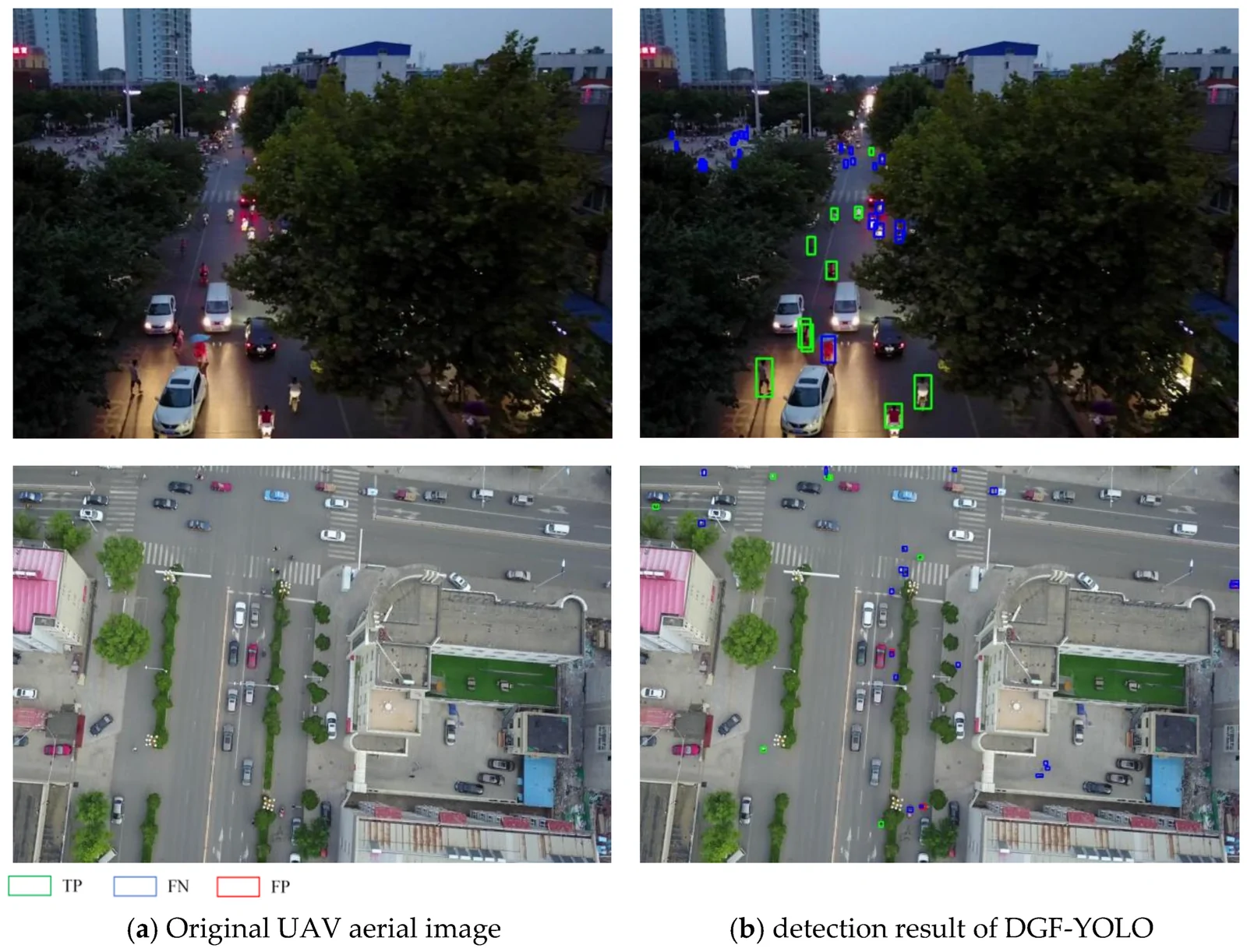
Detection results of DGF-YOLO in complex UAV aerial scenes. The first row (**a**) shows the detection results under low-light and tree-occlusion conditions, while the second row (**b**) shows the results in high-altitude top-down scenes containing extremely low-pixel pedestrians.

Although DGF-YOLO achieves favorable detection performance for small-scale pedestrians in UAV aerial imagery, certain limitations remain in complex scenarios. Figure 12 presents representative detection results under low-light, high-altitude top-down, and complex-background conditions. Green boxes indicate correctly detected targets, blue boxes indicate missed targets, and red boxes indicate false-positive detections.

As shown in Figure 12, under dusk or insufficient-illumination conditions, pedestrian targets exhibit low contrast against roads, tree shadows, and vehicle-light regions, resulting in substantially weakened target boundaries and local texture information. For such low-light targets, although the degradation-aware feature enhancement mechanism can improve the representation of certain weak-structure regions, missed detections may still occur because the input image itself contains insufficient discriminative information.

In addition, in high-altitude top-down scenes, some pedestrian targets occupy only a few pixels and appear as point-like or short-line structures. Their short-side structures may undergo substantial degradation even at the P2 feature level, making it difficult to adequately represent head–shoulder contours, body boundaries, and directional structures. Therefore, extremely low-pixel pedestrians remain one of the major challenges for the current model. Complex background elements, including tree occlusion, road textures, vehicle edges, and building shadows, can also affect detection performance. When small-scale pedestrians exhibit high similarity to background distractors in terms of appearance and local structure, the model may still produce false positives or missed detections.

These observations indicate that, although the proposed method alleviates structural degradation caused by network downsampling, it cannot fully recover missing local structural information under low-light, severe-occlusion, and extremely low-pixel conditions. Future work will focus on reducing the computational cost of the high-resolution P2 branch and associated feature-enhancement modules, while evaluating the method under broader UAV imaging conditions, particularly low-light and extremely low-pixel scenes.

## 5. Conclusions

This study presents DGF-YOLO for small-scale pedestrian detection in UAV imagery. The method uses a degradation-level criterion to analyze the short-side representation of pedestrians during feature downsampling and introduces a P2 detection layer according to the resulting distribution. DSA, DGA, and FSP are incorporated at the high-resolution feature level to model directional structures, degradation-sensitive regions, and local context.

On the VisDrone2019 pedestrian detection task, DGF-YOLO achieves 70.4% precision, 51.8% recall, 59.7% mAP50, and 26.9% mAP50-95. Compared with YOLOv12n, the corresponding improvements are 6.6, 7.4, 10.3, and 6.5 percentage points. The degradation-level-specific analysis also shows larger recall gains for targets undergoing degradation at relatively shallow feature levels.

These gains are accompanied by an increase in computation from 6.5 to 18.8 GFLOPs and a reduction in inference speed from 35.9 to 15.7 FPS. Extremely low-pixel, occluded, and poorly illuminated pedestrians also remain challenging. Future work will therefore focus on reducing the cost of high-resolution feature processing and evaluating robustness under broader UAV imaging conditions.

## Figures and Tables

**Table 1:** Experimental parameter settings for VisDrone2019-DET.

Parameter	Setup
Epochs	300
Input size	640 × 640
Batch size	4
Initial learning rate	1 × $10^{-2}$
Final learning rate	1 × $10^{-4}$
Optimizer	SGD
Weight decay	5 × $10^{-4}$

**Table 2:** Ablation Study Results of Different Improvement Modules.

Model	P2	DSA	DGA	FSP	P(%)	R(%)	mAP50 (%)	mAP50-95 (%)	Params (M)	GFLOPs	FPS
Baseline					63.8	44.4	49.4	20.4	2.50	6.5	35.9
A	√				67.0	49.7	56.4	24.2	2.70	12.3	25.1
AB	√	√			69.3	51.1	58.1	25.6	2.71	13.5	23.7
AC	√		√		67.4	50.7	58.5	25.8	2.78	15.6	19.2
AD	√			√	67.8	50.2	56.6	24.2	2.71	13.7	23.4
ABC	√	√	√		69.6	51.2	58.8	25.9	2.80	17.3	16.9
ABD	√	√		√	69.9	50.9	59.2	26.1	2.73	13.8	22.8
ABCD	√	√	√	√	**70.4**	**51.8**	**59.7**	**26.9**	2.81	18.8	15.7

√ indicates that the corresponding module is incorporated into the model. Bold values indicate the best performance among all evaluated methods.

**Table 3:** Sensitivity Analysis of the Degradation Auxiliary Loss Weight $\lambda_{deg}$

$\lambda_{deg}$	P(%)	R(%)	mAP50 (%)	mAP50-95 (%)
0	67.1	50.1	56.9	24.8
0.05	67.3	50.4	57.8	25.4
0.1	67.4	50.7	58.5	25.8
0.15	67.7	50.5	58.2	25.7
0.2	67.8	50.2	58.0	25.6

**Table 4:** Experimental results with different degradation thresholds τ.

τ	P(%)	R(%)	mAP50 (%)	mAP50-95 (%)
1.5	67.3	50.5	58.4	25.6
2.0	67.4	50.7	58.5	25.8
2.5	67.1	50.2	58.1	25.3
3.0	66.8	50.0	57.9	25.1

**Table 5:** Ablation Study Results for the Internal Structure of the DSA Module.

Model	P(%)	R(%)	mAP50 (%)	mAP50-95 (%)	Params (M)	GFLOPs
P2	67.0	49.7	56.4	24.2	2.702	12.3
P2 + 3×3 Conv	67.3	49.9	56.6	24.3	2.705	12.7
P2 + 3×1+1×3 Conv	68.1	50.4	57.4	24.8	2.706	12.8
P2 + 3×3+3×1 + 1×3 Conv	68.3	50.5	57.7	24.9	2.709	13.2
P2 + DSA	69.3	51.1	58.1	25.6	2.710	13.5

**Table 6:** Comparative Results of Different Attention Mechanisms with the P2 Detection Layer.

Model	P(%)	R(%)	mAP50 (%)	mAP50-95 (%)
P2	67.0	49.7	56.4	24.2
P2 + ECA	65.8	48.4	55.1	23.7
P2 + CA	66.2	48.7	55.5	23.9
P2 + EMA	66.5	48.9	56.2	24.1
P2 + CBAM	66.8	50.1	57.0	24.5
P2 + DGA	67.4	50.7	58.5	25.8

**Table 7:** Comparative Results of Different Convolutional Branches in the FSP Module.

Model	P(%)	R(%)	mAP50 (%)	mAP50-95 (%)
P2	67.0	49.7	56.4	24.2
P2 + DWConv	65.8	48.1	55.0	23.7
P2 + Dilated Conv	66.7	49.3	55.7	23.9
P2 + FSP	67.8	50.2	56.6	24.2

**Table 8:** Comparative Recall Results for Targets at Different Degradation Levels.

Model	P2-Deg	P3-Deg	P4-Deg	P5-Deg	Overall
YOLOv12n	39.8	57.2	61.6	63.3	44.4
YOLOv12n + P2	42.2	65.9	73.4	68.4	49.7
DGF-YOLO	**44.7**	**67.7**	**75.7**	**68.8**	**51.8**

Bold values indicate the best performance among all evaluated methods.

**Table 9:** Comparative Experimental Results.

Model	P(%)	R(%)	mAP50 (%)	mAP50-95 (%)	Params (M)	GFLOPs	FPS
YOLOX-Nano [34]	55.4	36.4	39.5	15.7	0.9	2.6	61.7
YOLOv5n	61.2	42.1	46.2	19.0	1.8	4.1	45.8
TPH-YOLOv5n [35]	62.2	43.0	47.9	19.5	2.6	8.3	32.0
YOLOv8n	62.9	43.6	48.6	20.1	3.2	8.7	30.4
YOLOv11n [36]	63.4	43.9	48.9	20.2	2.6	6.5	37.1
YOLOv12n(Baseline)	63.8	44.4	49.4	20.4	2.5	6.5	35.9
YOLOv12n + Sliced Training [37]	65.8	44.5	50.6	21.3	2.5	6.5	36.3
YOLOv12s	65.2	44.8	50.7	21.8	6.9	19.6	14.9
YOLOv12m	67.6	48.7	53.5	24.1	17.8	47.3	11.3
DGF-YOLO(Ours)	**70.4**	**51.8**	**59.7**	**26.9**	2.8	18.8	15.7

Bold values indicate the best performance among all evaluated methods.

**Table 10:** Generalization results on the full VisDrone2019-DET dataset.

Model	P(%)	R(%)	mAP50 (%)	mAP50-95 (%)	Params (M)	GFLOPs	FPS
YOLOX-Nano	38.2	27.0	27.5	14.1	0.9	2.6	60.2
YOLOv5n	41.4	30.3	29.8	15.7	1.8	4.1	44.7
TPH-YOLOv5n	42.9	32.5	32.7	16.5	2.6	8.3	31.3
YOLOv8n	43.4	32.1	33.0	16.9	3.2	8.7	29.4
YOLOv11n	44.6	32.8	34.1	17.2	2.6	6.5	35.2
YOLOv12n(Baseline)	44.9	33.4	34.7	17.5	2.5	6.5	30.7
YOLOv12n + Sliced Training	46.0	34.6	35.5	18.1	2.5	6.5	31.5
YOLOv12s	46.8	35.5	36.3	18.4	6.9	19.6	13.1
YOLOv12m	48.8	37.9	39.5	20.2	17.8	47.3	8.6
DGF-YOLO(Ours)	**51.7**	**40.4**	**43.8**	**22.3**	2.8	18.9	14.2

Bold values indicate the best performance among all evaluated methods.

## Data Availability

The VisDrone2019-DET dataset used in this study is publicly available from the official VisDrone repository. The converted single-class annotations and implementation details are available from the corresponding author upon reasonable request.
